# Muscle-specific Drp1 overexpression impairs skeletal muscle growth via translational attenuation

**DOI:** 10.1038/cddis.2014.595

**Published:** 2015-02-26

**Authors:** T Touvier, C De Palma, E Rigamonti, A Scagliola, E Incerti, L Mazelin, J-L Thomas, M D'Antonio, L Politi, L Schaeffer, E Clementi, S Brunelli

**Affiliations:** 1 E. Medea Scientific Institute, Bosisio, Parini, Italy; 2 Department of Clinical and Biomedical Sciences, Consiglio Nazionale delle Ricerche Institute of Neuroscience, L. Sacco University Hospital, Università di Milano, Milan, Italy; 3 Division of Regenerative Medicine, Stem Cells and Gene Therapy, San Raffaele Scientific Institute, Milan, Italy; 4 Department of Health Sciences, University of Milano-Bicocca, Monza, Italy; 5 Laboratoire de Biologie Moléculaire de la Cellule, Centre National de la Recherche Scientifique, Unité Mixte de Recherche 5239, IFR128, Université de Lyon, Equipe Différenciation Neuromusculaire, Ecole Normale Supérieure, Lyon Cedex 07, France; 6 Division of Genetics and Cell Biology, San Raffaele Scientific Institute, Milan, Italy; 7 Neuroradiology Group, Imaging Core, San Raffaele Scientific Institute, Milan, Italy

## Abstract

Mitochondrial fission and fusion are essential processes in the maintenance of the skeletal muscle function. The contribution of these processes to muscle development has not been properly investigated *in vivo* because of the early lethality of the models generated so far. To define the role of mitochondrial fission in muscle development and repair, we have generated a transgenic mouse line that overexpresses the fission-inducing protein Drp1 specifically in skeletal muscle. These mice displayed a drastic impairment in postnatal muscle growth, with reorganisation of the mitochondrial network and reduction of mtDNA quantity, without the deficiency of mitochondrial bioenergetics. Importantly we found that Drp1 overexpression activates the stress-induced PKR/eIF2*α*/Fgf21 pathway thus leading to an attenuated protein synthesis and downregulation of the growth hormone pathway. These results reveal for the first time how mitochondrial network dynamics influence muscle growth and shed light on aspects of muscle physiology relevant in human muscle pathologies.

Skeletal muscle growth and mitochondrial metabolism are intimately linked. In myogenic precursor cells, mitochondrial mass, mtDNA copy number and mitochondrial respiration increase after the onset of myogenic differentiation;^1, 2^ furthermore, postnatal development of fast-twitch muscle is accompanied by an increase in mtDNA copy number^3^ and muscle regeneration is impaired when mitochondrial protein synthesis is inhibited with chloramphenicol.^2, 4^ These observations suggest that a change in the mitochondrial metabolism is necessary for proper muscle development. During myogenesis and postnatal development, the shape of mitochondria is also remodelled:^3, 5, 6^ in an elegant mouse model with fluorescent mitochondria it was shown that in young mice mitochondria of the extensor digitorum longus (EDL) muscle are shaped as elongated tubules oriented along the long axis of the muscle fibre, whereas in adult mice mitochondria are punctuated and organised into doublets.^1^

Mitochondrial network morphology is controlled by the balance between fusion and fission. In mammals, three large GTPases are involved in mitochondrial fusion: mitofusins 1 and 2 (Mfn1 and Mfn2) participate in the early steps of mitochondrial outer-membrane fusion, whereas the optic atrophy 1 protein (Opa1) is essential for inner-membrane fusion.^7^ Mitochondrial fission is mediated by the evolutionarily conserved dynamin-related protein 1 (Drp1).^8^ In humans, mutations in Mfn2 and Opa1 cause two neurodegenerative diseases – Charcot–Marie–Tooth type 2 A and dominant optic atrophy, respectively – and a mutation in Drp1 has been linked to neonatal lethality with multisystem failure.^9, 10, 11^ Moreover, Drp1 expression was reported to increase in a model of cachexia^12^ and to contribute to muscle insulin resistance in obese and type 2 diabetic mice.^13, 14^

The importance of mitochondrial dynamics in muscle physiology has become increasingly clear. In skeletal muscle, mitochondria undergo fusion to share matrix content in order to support excitation–contraction coupling.^15^ The mitochondrial network is remodelled in atrophic conditions, and denervation and expression of fission machinery in adult myofibres is sufficient to cause muscle wasting.^16^ Moreover, mice lacking Mfn1 and 2 in fast-twitch muscles exhibit drastic growth defects and muscle atrophy before dying at 6–8 weeks of age.^3^ Animal models in which mitochondrial fission proteins are manipulated during skeletal muscle development are not yet available, but *in vitro* data demonstrate that regulation of Drp1 is critical for myogenesis: myoblasts differentiation requires nitric oxide-dependent inhibition of Drp1^6^ and pharmacological inhibition of Drp1 activity impairs myogenic differentiation.^17^

To explore *in vivo* the role of Drp1 and mitochondrial shape in the developing muscle, we generated a transgenic mouse line specifically overexpressing Drp1 in skeletal muscle during myogenesis. These mice display strong impairments in mitochondrial network shape and in muscle growth. We show that the mechanism responsible for the growth defect involves inhibition of protein synthesis and activation of the Atf4 pathway.

## Results

### Mice overexpressing Drp1 in muscle have reduced muscle mass and compromised exercise performance

**Figure 1:**
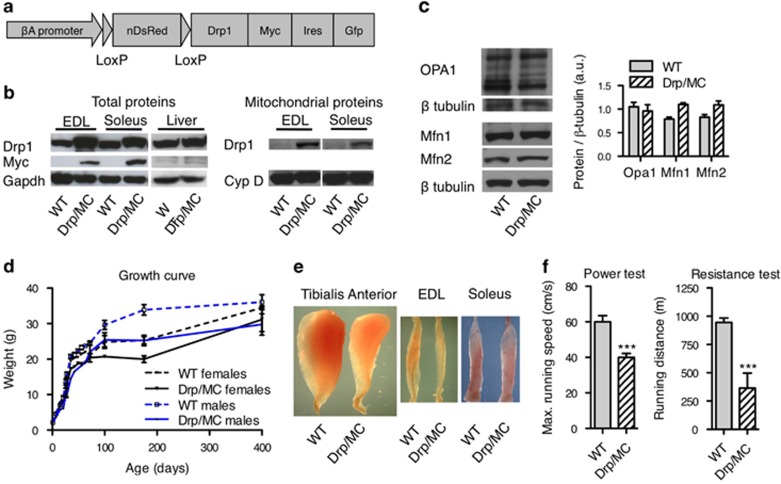
Mice overexpressing Drp1 (Drp/MC) in muscle have a severe reduction of muscle mass. (**a**) Transgene construct. (**b**) Left: Drp1 and Myc proteins were analysed by western blot in EDL and soleus muscles and liver of WT and transgenic animals, right: Drp1 expression in mitochondrial extract was analysed by western blot. Cyclophilin D (CypD) is used as a loading control. (**c**) Mitofusin 1 (Mfn1), Mitofusin 2 (Mfn2) and Opa1 expression was analysed by western blot. Quantification is given in the right chart (*n*=4 animals per genotype). (**d**) Growth curve of WT and Drp/MC animals (*n*=5 animals per group). (**e**) Pictures of WT and Drp/MC Tibialis anterior (TA), EDL and soleus muscles. (**f**) Speed and distance performed by P200 WT and transgenic mice on treadmill tests evaluating power and resistance (*n*=7 animals per genotype). Error bars represent S.E.M. ***P<0.001 *versus* wt

We generated a transgenic mouse that overexpresses Drp1 after Cre recombinase-mediated excision of floxed nuclear DsRed sequence (Figure 1a). Drp mice were crossed with MyoD^iCre^ transgenic mice, expressing Cre in myogenic cells.^18^ In double transgenic mice (Drp/MC), Drp1 protein was indeed overexpressed in muscles but not in the liver (Figure 1b). Analysis in EDL and soleus, respectively, a glycolytic and an oxidative muscle, showed that Drp1 expression increased in the mitochondrial protein fraction (Figure 1b). No significant changes were found in the levels of expression of the fusion proteins Mfn1, Mfn2 and Opa1 (Figure 1c). Drp/MC mice developed normally but displayed a growth defect starting 1 week after birth. At P100 body weight of transgenic animals was reduced by 20% compared with the wild-type (WT) mice (Figure 1d and Table 1). This defect was not because of a reduced food intake (Supplementary Table S1). Accordingly, levels of blood metabolites (triglycerides and glucose) and hormones (insulin, glucagon and leptin) did not differ significantly between Drp/MC and WT mice (Supplementary Table S1). Computerised tomography analyses in Drp/MC mice showed a significant reduction of total body volume and specific decreases of muscle and skeleton volumes (Supplementary Table S2). The length of the tibia bone was similar in WT and transgenic animals, indicating that Drp1 overexpression does not alter the whole-body growth: diminution of skeleton volume may be a consequence of decreased muscle and weight loading.^19^ Reduction of muscle mass was mainly because of a significant decrease in the weight of glycolytic muscles, such as tibialis anterior (TA), gastrocnemius and quadriceps (−50% *vs* WT), whereas the weights of oxidative muscles (soleus and diaphragm) were weakly affected (Table 1 and Figure 1e). This muscle phenotype strongly impacts on the locomotor performance of transgenic mice both in short-term high-intensity exercise and in long-term low-intensity running test (Figure 1f).

### Muscle-specific overexpression of Drp1 impairs postnatal skeletal muscle growth

**Figure 2:**
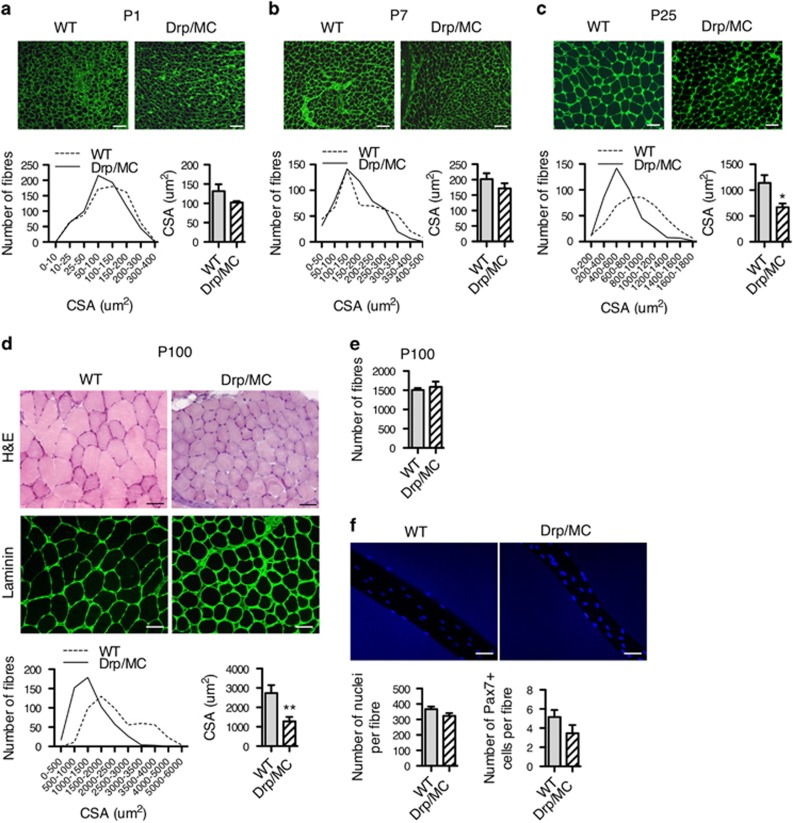
Muscle-specific Drp1 overexpression impairs postnatal muscle growth. (**a**, **b**, **c**) Representative images of immunofluorescence on TA sections from P1 (**a**), P7 (**b**) and P25 (**c**) mice by using a laminin-specific antibody. Original magnification x20, scale bar 50um. Frequency histogram showing the distribution of myofibre CSA in WT and Drp/MC TA muscles from P1 (**a**), P7 (**b**) and P25 (**c**) mice. Per condition, at least 500 fibres were counted. Mean myofibre CSA in WT and Drp/MC from P1 (**a**), P7 (**b**) and P25 (**c**) mice (*n*≥3 animals per genotype). (**d**) Representative sections of WT and Drp/MC TA muscles from P100 mice stained with H&E. Representative images of immunofluorescence on TA sections by using a laminin-specific antibody. Frequency histogram showing the distribution of myofibre CSA in WT and Drp/MC TA muscles from P100 mice. Mean myofibre CSA in WT and Drp/MC (*n*=3 animals per genotype). (**e**) Mean number of myofibres muscle (*n*=3 animals per genotype). (**f**) Mean number of myonuclei and Pax7 positive cells in WT and Drp/MC single fibres (*n*=4 preparations per genotype). Original magnification x20, scale bar: 50 *μ*m. Error bars represent S.E.M. **P<0.01; *P<0.05 *versus* WT

We investigated whether the phenotype observed in adult animals was due to defect in muscle development. At birth, Drp/MC newborns were indistinguishable from their control littermates, with normal gross morphology and myofibres cross-sectional area (CSA) (Figure 2a). One week after birth (P7), the mean CSA of Drp/MC myofibres was slightly reduced but not statistically different from that of WT controls (*n*=3 per genotype, *P*=0.10, Figure 2b). Myofibres CSA became significantly less in transgenic mice at P25 and P100 (−40 to –50%, Figures 2c and d). In adult transgenic mice, haematoxylin and eosin (H&E) staining showed a normal overall muscle morphology without inflammatory infiltrate and/or centrally nucleated fibres (Figure 2d). Likewise, we did not find alterations in the localisation and morphology of the neuromuscular junctions as indicated by the staining for the acetylcholine receptor and Sv2 (Supplementary Figure S1). Immunofluorescence (IF) for myosin heavy chains (MHC) 2A and 2B in the TA indicated that the myofibre typology of Drp/MC was unaffected (Supplementary Figure S2). The total number of TA myofibres, the mean number of myonuclei and the number of Pax7 positive satellite cells per fibre was similar in transgenic and WT mice (Figures 2e and f) suggesting that embryonic/fetal myogenesis occurs normally and that fusion of satellite cells to the pre-existing fibres during perinatal myogenesis is not impaired. Moreover muscle regeneration after cardiotoxin damage, a process that involves proliferation, fusion and differentiation of satellite cells, occurred in transgenic animals with the same time course as in WT counterparts (Supplementary Figure S3) thus confirming that satellite cells function is not impaired in Drp/MC mice.

In order to investigate the molecular pathways responsible for the reduced muscle growth, we analysed the activation of the ubiquitin–proteasome and the autophagy-lysosome systems. The expression of the ubiquitin ligases Atrogin-1/MAFbx, MuRF1 and Mul1 did not differ in developing and mature Drp/MC muscles, (Supplementary Figure S4). In muscles from Drp/MC and WT animals the levels of lipidated microtubule-associated protein-1 light chain 3 (LC3-II) and p62, two markers of autophagy (Supplementary Figure S4), were similar, as well as the phosphorylation levels of AMPK, the cell sensor of energy balance (Supplementary Figure S4).

Taken together, these data suggest that embryonic, fetal and perinatal myogenesis occur properly in transgenic animals and that Drp1 overexpression impairs the increase in myofibres size during the perinatal/postnatal period without changes in satellite cells function or activation of catabolic pathways.

### Postnatal mitochondrial network remodelling is impaired in Drp/MC muscle

**Figure 3:**
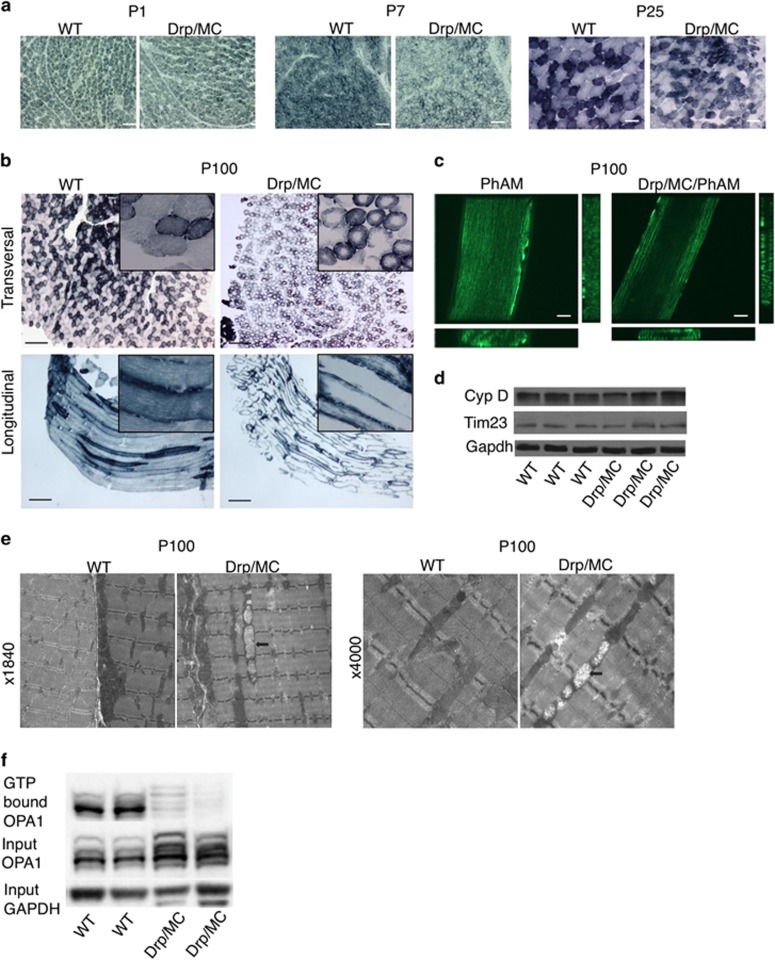
Muscle-specific Drp1 overexpression induces a drastic remodelling of mitochondrial network. (**a**) SDH staining of transversal sections from WT and Drp/MC from P1, P7, P25 TA muscles. Original magnification × 20, scale bar: 50 *μ*m. (**b**) SDH staining of transversal and longitudinal sections from WT and Drp/MC P100 TA muscle. Low magnification, × 4; high magnification, × 40. (**c**) Representative confocal images of mitochondrial network in PhAM and Drp/MC/PhAM TA myofibres from P100 animals. Transversal and longitudinal optical sections are also shown. Original magnification x40, scale bar: 10 *μ*m. (**d**) Western blot analysis of Tim23 and Cyclophilin D (CypD) protein expression levels in mitochondrial protein lysates from quadriceps of adult WT and Drp/MC mice. Gapdh was used as internal control. (**e**) Electron microscopy analysis of TA muscles from P100 Drp/MC and WT mice. Arrows point to swollen mithocondria, with none or fewer than normal cristae. (**f**) Western blot analysis of Opa1 GTPase activity after pull-down experiments using GTP-conjugated beads

We next analysed whether Drp1 overexpression affects the activity and distribution of mitochondria by staining muscle TA sections for succinate dehydrogenase (SDH) activity. At P1 no differences were observed between Drp/MC and WT animals (Figure 3a). In Drp/MC P7 muscles, the intensity of SDH staining, a proxy for oxidative capacity, was slightly reduced (Figure 3a) and, at P25, it was found in punctuated units in most fibres and some were completely devoid of oxidative capacity in their core (Figure 3a). In adult Drp/MC muscles (P100), the oxidative capacity of mitochondria was found restricted to the fibre periphery (Figure 3b). Similar results were observed with cytochrome oxidase (COX) staining (Supplementary Figure S5). To visualise specifically the structure and not only the activity of the mitochondria, we crossed Drp/MC transgenic mice with PhAM transgenic mice, a line with photo-activatable mitochondria.^1^ In control PhAM TA, we observed the typical striated distribution of inter-myofibrillar and sub-sarcolemmal mitochondria. By contrast, Drp/MC/PhAM muscles displayed a severe remodelling of the mitochondrial network, lacking most of the internal inter-myofibrillar mitochondria (Figure 3c and Supplementary Movies 1 and 2). Electron microscopy analysis of TA myofibres of Drp/MC mice revealed an overtly normal myofibre ultrastructure, still the mitochondrial network was clearly altered, with virtual absence of the inner pool of inter-myofibrillar mitochondria. In addition, while many mitochondria showed a normal structure, some appeared swollen, with no or fewer than normal cristae (Figure 3e). Accordingly we found that GTP-binding activity of Opa1 that resides in the inner mitochondrial membrane was largely decreased in Drp/MC muscles (Figure 3f).

This mitochondrial network alteration was not due to changes in mitochondrial number or turnover: the mitochondrial mass assessed by the protein levels of Tim23 and Cyclophilin D, did not differ between WT and transgenic animals (Figure 3d). Accordingly, protein levels of PGC1*α*, a master regulator of mitochondrial biogenesis were similar (Supplementary Figure S6A). In addition, the levels of membrane-anchored LC3 (LC3-II) was comparable in WT and Drp/MC muscles and no co-localisation between phagosomes and mitochondria was observed (Supplementary Figure S6B and S6C), indicating that mitophagy was not triggered.

Overall these data suggest that Drp1 overexpression affects the mitochondrial structure and impairs the normal remodelling of the mitochondrial network in growing muscle by redistributing mitochondria close to the sarcolemma and/or myonuclei.

### Mitochondrial DNA content is reduced and a mitochondrial unfolded protein response (mtUPR) is activated in Drp/MC muscles

**Figure 4:**
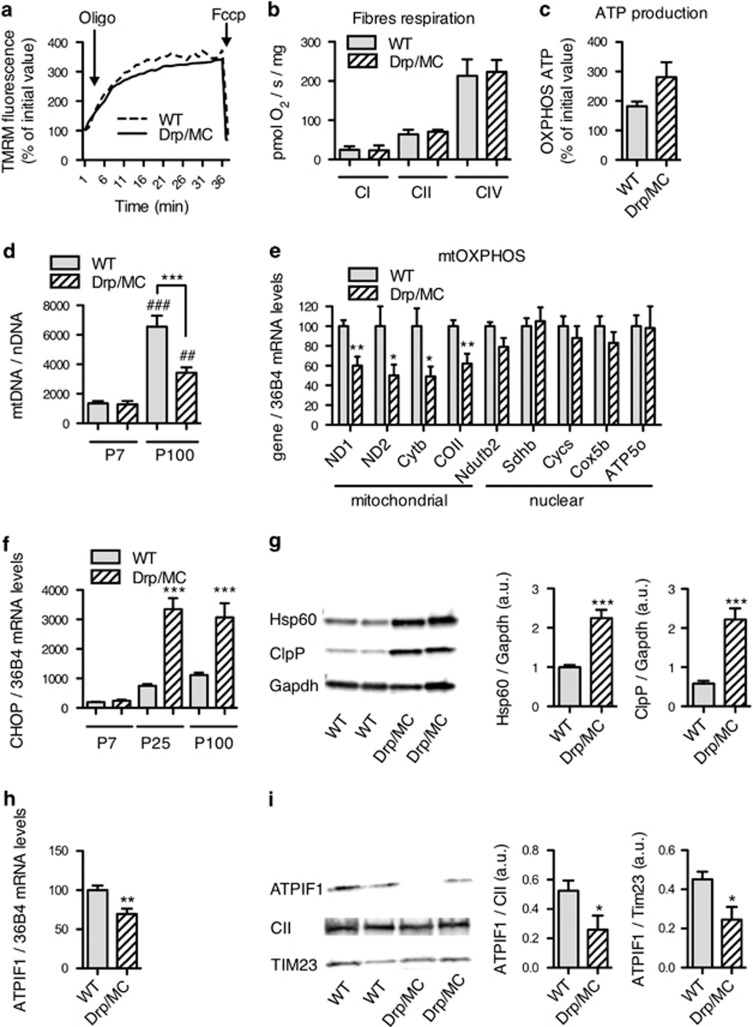
Mitochondrial bioenergetics remained unchanged but mtDNA content is reduced and a mtUPR response is activated in Drp/MC muscle. (**a**) Fibres were loaded with the mitochondrial potentiometric dye TMRM (25 nM) and the fluorescence was recorded for 40 min. The arrows indicate the time of addition of oligomycin (Oligo; 5 *μ*M) and FCCP (4 *μ*M). (**b**) Mitochondrial respiration in permeabilised TA myofibres was measured by using high-sensitivity respirometer (*n*=8 animals per genotype); complex I (CI), complex II (CII) and complex IV (CIV). (**c**) Isolated mitochondria from TA muscle were incubated with luciferin-luciferase and the ATP generated through oxidative phosphorylation was measured (*n*=9 animals per genotype). (**d**) Mitochondrial DNA (mtDNA) content in TA muscle from P7 and P100 animals (*n*=8 per genotype). (**e**) RT-qPCR analysis of mtDNA and nuclear DNA encoded respiratory chain subunits in P100 quadriceps muscle. (**f**) RT-qPCR analysis of Chop mRNA levels in quadriceps from P7, P25 and P100 WT and Drp/MC mice (*n*=8 per genotype). (**g**) Western blot analysis of Hsp60 and ClpP protein expression levels in quadriceps from adult WT and Drp/MC mice (*n*=8 per genotype). Actin was used as control. (**h**) RT-qPCR analysis of Atpif1 mRNA levels in quadriceps from P100 WT and Drp/MC mice (*n*=8 per genotype). (**i**) Western blot analysis of ATPIF1 protein expression levels in quadriceps from adult WT and Drp/MC mice (*n*=8 per genotype). Tim23 and Complex II (CII) were used as control. Error bars represent S.E.M. *P<0.05, **P<0.01, ***P<0.001 *versus* WT; ^##^*P*<0.01, ^###^*P*<0.001 *versus* P7 mice

To investigate specifically the mitochondrial functionality in Drp/MC muscles, we measured their membrane potential in myofibres from WT and Drp/MC gastrocnemius by using the fluorochrome tetramethyl rhodamine methyl ester (TMRM). In WT fibres, we observed the expected pattern with functional mitochondria arranged in a stereotypic manner in relation with the sarcomeric unit. In Drp/MC fibres, functional mitochondria were preferentially localised close to myonuclei (Supplementary Figure S7). We tested whether mitochondria of Drp/MC muscle display a latent dysfunction, that is, masked by the ATP synthase consuming ATP to maintain the mitochondrial membrane potential.^20^ Administration of the ATP synthase inhibitor oligomycin fibres increased the mitochondrial membrane potential in both WT and Drp/MC muscles suggesting that Drp/MC mitochondria are healthy (Figure 4a). Consistently, the rates of oxygen consumption, measured with substrates targeting complex I, II and IV in saponin-skinned fibres by oxygraphy, were similar in WT and Drp/MC mice (Figure 4b). Similarly, we found no differences in ATP levels produced through oxidative phosphorylation in isolated TA mitochondria (Figure 4c). These data indicate that Drp1 overexpression does not alter mitochondrial bioenergetics.

We then assessed whether Drp1 overexpression affects the increase in mtDNA that accompanies postnatal muscle development.^3^ As expected, we observed an increase in the mtDNA copy number between P7 and P100 mice; this increase, however, was 40% lower in Drp/MC mice with respect to WT mice (Figure 4d). Consistently, we observed a decrease in mRNA levels of the subunits of the electron transport respiratory chain encoded by mtDNA. No differences were found for subunits encoded by nuclear DNA (Figure 4e).

Since low-mtDNA amount induces a mitochondrial stress and a subsequent mitochondrial mtUPR upregulation,^21^ we measured the expression of mtUPR markers. mRNA levels of the C/EBP homologous protein (Chop), a master gene involved in mtUPR response^22^ increased significantly in Drp/MC quadriceps during the postnatal period (Figure 4f). Mitochondria-specific chaperonin 60 (Hsp60) and the protease ClpP, both hallmarks of the mtUPR were upregulated in quadriceps (Figure 4g), but not in liver and diaphragm (Supplementary Figure S8), of Drp/MC mice. Downregulation of the ATP synthase inhibitor ATPIF1 has been shown to ameliorate mitochondrial respiratory chain dysfunction;^23^ mRNA and protein levels of ATPIF1 were significantly reduced in Drp/MC quadriceps (Figures 4h and i). Overall these data suggest that Drp1 overexpression reduces mtDNA quantity and activates a specific protective response involving mitochondrial chaperones and ATPIF1, which successfully preserves the mitochondrial function.

We verified whether mtUPR was associated to a classical UPR induced by ER stress. We found no alterations in mRNA levels of the ER-resident heat shock protein 70 (HSP70) family members BIP/GRP78 and GRP94, nor splicing of X box binding protein 1 (XBP-1) mRNA in Drp/MC muscles (Supplementary Figure S9). Thus Drp1 overexpression induced specific mitochondrial stress and mtUPR.

### Muscle-specific Drp1 overexpression inhibits protein synthesis and induces Atf4 pathway

**Figure 5:**
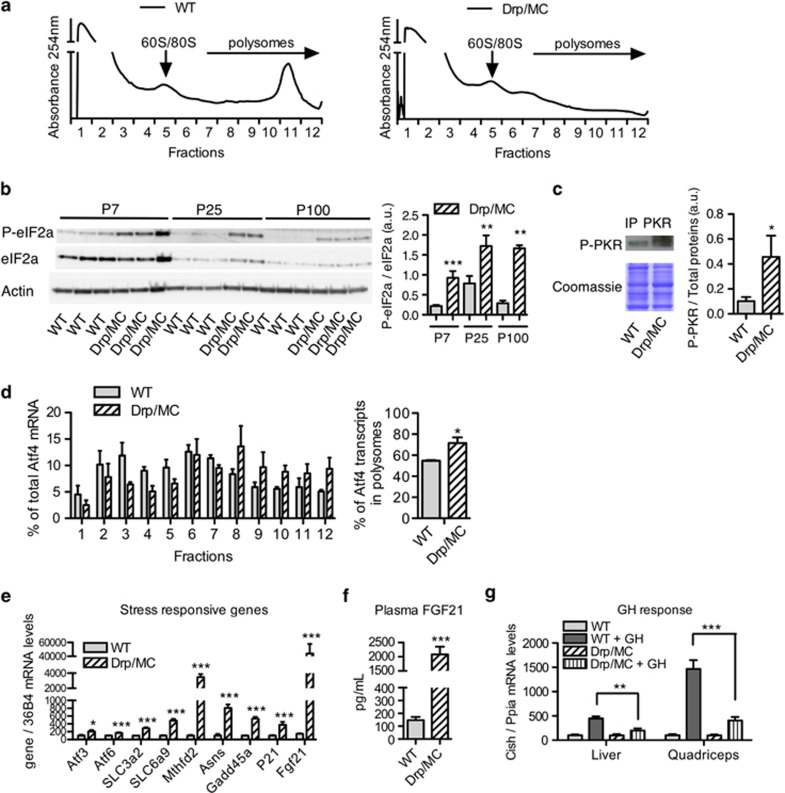
eIF2*α*/Atf4 pathway is activated in Drp/MC muscle. (**a**) Polysome profile in quadriceps from young (P40) WT and Drp/MC mice. Lysates were analysed by centrifugation in a 10–50% sucrose gradient, and the profiles were measured by absorbance at 254 nm. The panel is representative of three independent experiments. (**b**) Representative western blot analysis of phosphorylated and total eIF2*α* protein expression levels in quadriceps from P7, P25 and adult (P100) WT and Drp/MC mice. Quantification is given in the right chart (*n*=5 per genotype). (**c**) Western blot analysis of phosphorylated PKR protein levels after immunoprecipitation of total PKR protein from gastrocnemius protein lysate of adult (P100) WT and Drp/MC mice (*n*=3 per genotype). (**d**) Atf4 mRNA distribution in the fractions collected from the sucrose gradients was determined by RT-qPCR. Percentage of Atf4 transcripts present in polysomal fractions (6–12) (*n*=3 per genotype). (**e**) RT-qPCR analysis of representative stress-response genes mRNA levels in quadriceps from adult WT and Drp/MC mice (*n*=8 per genotype). (**f**) Plasma levels of Fgf21 in adult WT and Drp/MC mice (*n*=11 per genotype). (**g**) RT-qPCR analysis of Cish mRNA levels in quadriceps from adult WT and Drp/MC mice 4 h after GH treatment (1.5 mg/kg i.p., *n*=7 per group). Error bars represent S.E.M. *P<0.05, **P<0.01, ***P<0.001 *versus* WT

In order to investigate the mechanisms responsible for the reduced postnatal muscle growth, we monitored protein synthesis by analysing polysome profile in control and Drp/MC P40 muscles by using sucrose gradient centrifugation (Figure 5a). This allowed to separate polysomes from monosomes, ribosomal subunits and messenger ribonucleoprotein particles (mRNPs) and enabled discrimination between efficiently translated (associated with heavy polysomes) and poorly translated (associated with light polysomes) mRNAs. In Drp/MC muscles, we observed a huge reduction of medium and heavy polysomes (fractions 8–12), concomitant with elevated levels of free ribosomes and light polysomes, indicating that translation initiation is severely altered.

We also verified whether the AKT/GSK3*β*/mTOR pathway, a crucial regulator a protein synthesis, was modulated. Although levels of both total and phosphorylated forms of 4EBP1 and S6 were increased in Drp/MC muscles, possibly because of the alteration of protein synthesis observed above, the phosphorylation levels of AKT and GSK3*β* were similar in WT and Drp/MC muscles. (Supplementary Figure S10), suggesting that the mTOR pathway is not involved in the translation impairment observed.

Eukaryotic initiation factor 2 alpha (eIF2α) is another crucial regulator of protein translation. Its phosphorylation at Ser-51 reduces the global translation initiation while enhancing the translation of selected mRNAs, such as those activating transcription factor (Atf4), a master gene of the integrated stress response.^24^ Mitochondrial stress was recently shown to activate the eIF2*α* stress-response pathway in a double-stranded RNA-activated protein kinase (PKR) dependent way.^25^ We found that phosphorylation levels of eIF2*α* were highly increased in transgenic muscles (Figure 5b), as well as expression of the phosphorylated form of PKR (Figure 5c) suggesting that Drp1-mediated mitochondrial stress activates PKR, which in turn phosphorylates eIF2*α*. Consistently, we observed in transgenic muscles a substantial shift of Atf4 and Chop transcripts to the large polysome fractions^26^ (Figure 5d and Supplementary Figure S11A). Conversely there is a trend of MHC transcripts being decreased associated with larger polysomes, in agreement with the reduced content of myosin proteins (Supplementary Figures S11B-C). As a control, we measured the levels of 36B4 mRNA and found that this transcript was insensitive to the integrated stress response induced by Drp1 overexpression (Supplementary Figure S11D).

Atf4 activates the transcription of many target genes in muscle that may interfere with muscle growth.^27^ Indeed growth arrest and DNA-damage-inducible protein alpha (Gadd45a) and fibroblast growth factor 21 (Fgf21) were upregulated in transgenic muscles (Figure 5e). In mouse models of mitochondrial diseases as well as in human patients, skeletal muscle is known to secrete high amount of Fgf21.^28^ In adult Drp/MC mice, serum Fgf21 concentration was 14-fold higher compared with control mice (WT 148±25 *vs* Drp/MC 2084±269 pg/ml) (Figure 5f). Fgf21 and Gadd45a are both known to repress growth hormone (GH)-dependent pathways:^29, 30, 31^ we treated adult WT and Drp/MC mice with hGH and measured mRNA levels of the GH target gene cytokine-inducible inhibitor of signalling (Cish) (Figure 5g). Induction of Cish was significantly reduced in both liver and quadriceps of Drp/MC mice, suggesting that muscle-specific DRP1 overexpression leads to a global reduction of GH sensitivity. Moreover, the expression of several genes involved in GH action was deregulated in Drp/MC quadriceps: expression of growth hormone receptor (Ghr) and its downstream effector Jak2 were strongly downregulated, whereas Leprotl1, a gene that negatively regulates Ghr presence at the cell surface,^32^ was upregulated (Supplementary Figure S12). Altogether these data suggest that Drp1 overexpression triggers an adaptive stress response that prevents normal fibre growth by attenuating general protein translation and downregulating growth hormone anabolic pathway.

## Discussion

In this study, we demonstrate that skeletal muscle-specific overexpression of the mitochondrial fission protein Drp1 induces a reorganisation of the mitochondrial network and impairs postnatal muscle development. Drp1 induces a persistent activation of eIF2*α*/Atf4 stress-response cascade that interferes with muscle anabolism by inducing a general depression of translation and by reducing growth hormone signalling.

In mature skeletal muscle, mitochondria are tightly packed and arranged in a stereotypic manner in relation to the sarcomeric unit.^33^ This complex organisation is reached by 30 days after mouse birth and depends on mitochondrial fusion proteins.^1, 3^ In muscle overexpressing Drp1 we observed that postnatal mitochondrial network remodelling does not occur properly, and that mitochondrial distribution is drastically redistributed towards the myonuclei. This kind of rearrangement has been observed in cellular models defective in fission or fusion proteins expression.^5, 34, 35^ Little, however, is known on the role of this process apart from a possible calcium buffering activity at the plasmalemma^34^ or regulation of gene transcription *via* increased nuclear ROS levels.^36^ Drp1 overexpression also prevents the normal increase of mtDNA quantity occurring in the postnatal period,^37^ suggesting a link between mitochondrial shape and mtDNA maintenance/replication.

Although Drp1 overexpression modulates mitochondrial shape and DNA content, it does not affect mitochondrial polarisation, cellular respiration and ATP production. Indeed, activation of the cellular fuel gauge AMPK and of the anabolic/catabolic pathways controlled by energy balance (ubiquitin–proteasome, autophagy, AKT-mTOR) is not altered in Drp/MC muscles. Romanello *et al.*,^16^ reported that electroporation of Drp1 and Fis1 in adult muscle promotes mitochondrial fragmentation, mitophagy, activation of AMPK and muscle atrophy. In our model, Drp1 overexpression is probably lower than in their model: the resulting more moderate mitochondrial stress could therefore be handled more easily by the muscle, and no energy imbalance becomes unmasked, also because of the reduced muscle growth. It is also possible that mitochondrial alterations triggered by Drp1 overexpression are compensated by upregulation of ATP synthase activity *via* reduction of ATPIF1 levels,^23^ that is decreased in Drp/MC mitochondria.

In Drp/MC muscles, we observed activation of the eIF2*α* pathway, which allows the cells to adapt to various stresses by downregulating protein translation and inducing Atf4, a transcriptional activator of the integrated stress response.^24^ Interestingly, this pathway is induced when myoblasts are pushed towards differentiation and inhibits transiently the myogenic programme.^38^ In Drp1 overexpressing muscles, eIF2*α*/Atf4 pathway is activated during the whole postnatal muscle development, prior to the appearance of muscle phenotype (around P25). Thus it is likely that activation of this pathway is a cause rather than a consequence of altered muscle development. Other studies have demonstrated a link between mitochondria-shaping proteins and eIF2*α* phosphorylation. Genetic ablation of Mfn2 (but not Mfn1) induces an eIF2*α* phosphorylation as a result of ER stress and PERK activation.^39, 40^ In contrast, phosphorylation of eIF2*α* in Drp/MC muscle is likely induced by specific mitochondrial stress, because we observed an on-going mtUPR response and no changes in the expression of ER stress markers. Moreover, we found increased levels of phosphorylated PKR, a cytosolic eiF2*α* kinase described to be activated by specific mitochondrial stress.^25^ The link between Drp1-mediated mitochondrial alteration and PKR activation remains to be investigated.

As expected in conditions of high P-eIF2*α* levels, we observed a general depression of translation and a reduced content of MHC. In cachectic cancer patients there is an inverse correlation between MHC expression and the phosphorylation levels of eIF2*α*,^41^ and tumour-derived factors such as proteolysis inducing factor (PIF) produce a depression in protein synthesis in murine myotubes through autophosphorylation of PKR and increased phosphorylation of eIF2*α*.^42^ Increased phosphorylation of PKR/eIF2*α* was also observed in muscles from diabetic mice.^43^ Since Drp1 expression levels increases in cachexia and type 2 diabetes models,^12, 13^ it is possible that some of the events that we observed in Drp/MC muscle are also responsible for the depression in protein synthesis observed in these pathologies.

In Drp/MC muscles, we also observed preferential translation of Atf4 and upregulation of its target genes, events consistent with the phosphorylation of eIF2*α*. Atf4 overexpression in adult muscle was shown to reduce myofibres size and some of its downstream target genes may interfere with muscle development.^27^ In particular Fgf21, a sensitive marker of muscle mitochondrial diseases,^28, 44^ was strongly upregulated in skeletal muscles and in blood of Drp/MC mice. Fgf21 was recently shown to inhibit growth hormone (GH) action by increasing Leprotl1 expression.^29^ Accordingly, muscles of Drp/MC mice displayed a reduced GH sensitivity, upregulation of Leprotl1 gene expression and a downregulation of genes involved in GH signalling. These alterations may interfere with the growth-promoting action of GH during the muscle development.^45^

In conclusion, our study shows that mild alteration of balance between fusion and fission impairs specifically muscle growth that is dependent on protein synthesis. This is the first demonstration that increased Drp1 levels induces a specific mitochondrial stress response and inhibits cytosolic protein translation. It thus appears that Drp1 and its dependent signalling are at the crossroad of pathways critical for muscle homeostasis, such that their imbalance is observed in diverse pathological conditions of the muscle. Manipulation of mitochondrial dynamics and of eIF2*α* pathway may therefore be useful in developing therapeutic interventions for myopathies characterised by protein synthesis depression.

## Materials and Methods

### Generation of skeletal muscle-specific Drp1 transgenic mice

To generate the Drp1 transgenic mice Drp1 ORF was inserted into the piGAP vector.^46^ Drp transgenic mice were crossed with heterozygous MyoD^iCre^ knockin mice.^18^ Details on the cloning and animal experimentation are provided in Supplemental Experimental Procedures. All experiments conformed to Italian law and were performed under internal regulations for animal care and handling (San Raffaele Institute IACUC 355 and 489).

### Western blot analysis and Immunoprecipitation

Muscle and liver tissues were homogenised in lysis buffer containing 20 mM HEPES pH 7.4, 150 mM NaCl, 2% sodium dodecyl sulphate (SDS), protease and phosphatase inhibitor mixture (Roche, Basel, Switzerland) and centrifuged at 1000 × *g* for 10 min at 4 °C to discard cellular debris. Western blot analyses were performed as described.^6^ For PKR expression studies, gastrocnemius muscle from adult WT and transgenic mice were homogeneised in lysis buffer containing 20 mM HEPES pH 7.4, 150 mM NaCl, 1 mM EDTA, 10% glycerol, 1% Triton X100, protease and phosphatase inhibitor mixture (Roche). A total of 1.5 mg of lysate was incubated overnight at 4 °C with 4 *μ*g of antibody against total PKR. The complexes were pelleted with protein A–Sepharose (Invitrogen, Carlsbad, CA, USA), and then separated by SDS-PAGE with an antibody against PKR. Details on the antibodies are provided in Supplementary Table S3.

### Histology and immunofluorescence

Serial muscle sections were stained with H&E (Sigma-Aldrich, St. Louis, MO, USA) or succinic dehydrogenase staining (Bio-Optica, Milano, Italy). IF staining was performed as in Rigamonti *et al.*^47^ Details on the procedures and the antibodies are provided in Supplementary data and Supplementary Table S3.

### RT-qPCR analyses

Total RNA was extracted from muscles by using TRIzol reagent (Invitrogen). Total RNA (1 *μ*g) was reverse transcribed with random hexameric primers and MultiScribe reverse transcriptase (Applied Biosystems, Warrington, UK). cDNAs were quantified by quantitative real-time PCR on a MX 3000 apparatus (Stratagene, La Jolla, CA, USA) by using specific primers. Primer sequences are provided in Supplementary data and Supplementary Table S4. PCR amplification was performed in a volume of 20 *μ*l containing 5 ng cDNA, 300 nM of each primer, GoTaq qPCR Master Mix (Promega, Madison, WI, USA). Gene expression changes were normalised to 36B4 or Ppia gene expression by using the ΔΔCT method. XBP1 splicing assay was performed with TaqMan PerkinElmer assays (Perkin Elmer, Boston, MA, USA), as described.^48^

### Electron transmission microscopy

Muscle tissue blocks were fixed in 2.5% glutaraldehyde in 0.1 M phosphate buffer (pH 7.4). Post-fixation was performed in 1% OsO4 in 0.1 M cacodylate buffer (pH 7.4) supplemented with 1.5% K4[Fe(CN)6]. Subsequently, samples were dehydrated and embedded in epon. Ultrathin sections were examined by using a LEO 912AB electron microscope.

### Assay of GTPase activity

Forty milligram of quadriceps muscle was homogenised in RIPA buffer containing 50 mM Tris-HCl, 150 mM NaCl, 1% NP-40, 1% sodium deoxycholate, SDS 0,1% and the protease inhibitor cocktail (pH 7.5). After centrifugation at 12 000 r.p.m. for 5 min, the supernatant was recovered and protein concentration was quantified. 300 *μ*g of proteins were incubated in a reaction buffer (50 mM Tris-HCl, 150 mM NaCl, 1 mM MgOAc2, 1 mM dithiothreitol, the protease inhibitor cocktail, pH 7.5) with 50 *μ*l GTP-agarose beads (Sigma) at 30 °C for 1 h. The beads were centrifuged at 3000 r.p.m. for 5 min and washed three times in the reaction buffer and the proteins were resolved on SDS-PAGE and analysed by immunoblotting by using anti-Opa1 antibody.

### Quantification of mitochondrial DNA (mtDNA) copy number per nuclear DNA (nuDNA)

Total genomic DNA was isolated by using the QIAamp DNA Micro kit (Qiagen, Venlo, Netherlands). The amount of mtDNA per nuclear genome was quantified by qPCR by using specific primers for cytochrome b, ND2, 28 S and Pecam. Primers sequences are provided in Supplementary Table S4. Quantification of the relative copy number of mtDNA per nuDNA was analysed by using the ΔCT method.

### Mitochondrial membrane potential analysis

Gastrocnemius myofibres were loaded with 25 nM tetramethyl rhodamine methyl ester (TMRM, Molecular Probes, Eugene, OR, USA) for 30 min at 37 °C. Sequential images of TMRM fluorescence were acquired every 60 s with a x40 objective on confocal microscope (UltraView, Perkin Elmer), with or without the administration of the ATP synthase inhibitor oligomycin (5 uM) (Sigma-Aldrich) or the protonophore carbonyl cyanide *p*-trifluoro-methoxyphenylhydrazone (FCCP, 4 uM) (Sigma-Aldrich).

### Preparation of isolated mitochondria

Mitochondria from TA muscle were obtained as described^49^ with slight modifications. Details are provided in Supplemental Experimental Procedures.

### Measurement of ATP formation

ATP concentration was determined by using the luciferin/luciferase method as in^6^ and details are provided in Supplemental Experimental Procedures.

### Oxygen consumption on permeabilised fibres

Oxygen consumption on the isolated TA fibres was performed as in.^6^ Details of the procedure are described in Supplemental Experimental Procedures.

### Polysome analysis and translational control

Polysome isolation on sucrose gradients (10–50%) and RNA extraction were performed as previously described^50^ by using a pool of two frozen quadriceps from P40 control or Drp/MC mice (*n*=3). Same muscle protein amount was layered on the gradients. Synthetic luciferase reporter mRNA (3 ng) was added in each fraction before extraction and was used as an internal control. Reverse transcription and qPCR were performed as above but the measurements were normalised to luciferase abundance.

### Statistics

Statistical significance of variations among the different experimental groups was determined by two-tailed Student's *t*-test (Prism 5, GraphPad Software). *P* -values <0.05 were considered significant.

## Figures and Tables

**Table 1:** Tissue measurements in P100 wild-type and Drp/MC mice

	**Females**		**Males**	
	**WT**	**Drp/MC**	**WT**	**Drp/MC**
Body weight (g)	25±2	21±0**	30±1	25±2**
Tibialis anterior (mg)	47±3	22±1***	54±2	31±4***
EDL (mg)	10±0	5±1***	12±1	6±1***
Soleus (mg)	10±0	7±1*	9±0	9±1
Gastrocnemius (mg)	181±8	103±6***	187±7	117±14***
Quadriceps (mg)	179±13	93±4***	197±13	122±18***
Diaphragm (mg)	86±11	73±7	87±3	80±4
Heart (mg)	137±8	117±3	147±8	151±11
Perigonadal AT (mg)	603±130	418±44	658±102	460±80
Liver (mg)	1409±156	1200±61	1549±102	1566±100
Kidney (mg)	399±32	361±12	503±28	505±33
Spleen (mg)	131±16	116±5	103±7	113±18

Data are given ± S.E.M. N⩾4 mice per group. *T*- test *vs* WT, *P<0.05 **P<0.01 ***P<0.001

## Abbreviations

Atf4: activating transcription factor-4
Cish: cytokine-inducible inhibitor of signal
Chop: C/EBP homologous protein
CSA: cross-sectional area
Drp1: dynamin-related protein 1
EDL: extensor digitorum longus
eIF2: Eukaryotic initiation factor 2
ER: endoplasmic reticulum
Fgf21: fibroblast growth factor 21
Gadd45a: growth arrest and DNA-damage-inducible protein DNA
H&E: haematoxylin and eosin
HSP70: heat shock protein 70
IF: Immunofluorescence
Mfn: mitofusin
mtDNA: mitochondrial DNA
MHC: myosin heavy chains
mRNPs: messenger ribonucleoprotein particles
mtUPR: mitochondrial unfolded protein response
Opa1: optic atrophy 1 protein
PGC1*α*: Peroxisome proliferator-activated receptor gamma coactivator 1-alpha
TA: tibialis anterior
PKR: double-stranded-RNA-activated protein kinase
TMRM: tetramethyl rhodamine methyl ester
XBP-1: X box binding protein 1.

## References

[bib1] 1Pham AH, McCaffery JM, Chan DC. Mouse lines with photo-activatable mitochondria to study mitochondrial dynamics. Genesis 2012; 50: 833–843. 22821887 10.1002/dvg.22050 PMC3508687

[bib2] 2Wagatsuma A, Sakuma K. Mitochondria as a potential regulator of myogenesis. Sci World J 2013; 2013: 1–9. 10.1155/2013/593267 PMC3574753 23431256

[bib3] 3Chen H, Vermulst M, Wang YE, Chomyn A, Prolla TA, McCaffery JM et al. Mitochondrial fusion is required for mtDNA stability in skeletal muscle and tolerance of mtDNA mutations. Cell 2010; 141: 280–289. 20403324 10.1016/j.cell.2010.02.026 PMC2876819

[bib4] 4Wagatsuma A, Kotake N, Yamada S. Muscle regeneration occurs to coincide with mitochondrial biogenesis. Mol Cell Biochem 2011; 349: 139–147. 21110070 10.1007/s11010-010-0668-2

[bib5] 5Bach D, Pich S, Soriano FX, Vega N, Baumgartner B, Oriola J et al. Mitofusin-2 determines mitochondrial network architecture and mitochondrial metabolism. A novel regulatory mechanism altered in obesity. J Biol Chem 2003; 278: 17190–17197. 12598526 10.1074/jbc.M212754200

[bib6] 6De Palma C, Falcone S, Pisoni S, Cipolat S, Panzeri C, Pambianco S et al. Nitric oxide inhibition of Drp1-mediated mitochondrial fission is critical for myogenic differentiation. Cell Death Differ 2010; 17: 1684–1696. 20467441 10.1038/cdd.2010.48 PMC3050583

[bib7] 7Chan DC. Fusion and fission: interlinked processes critical for mitochondrial health. Annu Rev Genet 2012; 46: 265–287. 22934639 10.1146/annurev-genet-110410-132529

[bib8] 8Otera H, Ishihara N, Mihara K. New insights into the function and regulation of mitochondrial fission. Biochim Biophys Acta 2013; 1833: 1256–1268. 23434681 10.1016/j.bbamcr.2013.02.002

[bib9] 9Delettre C, Lenaers G, Griffoin JM, Gigarel N, Lorenzo C, Belenguer P et al. Nuclear gene OPA1, encoding a mitochondrial dynamin-related protein, is mutated in dominant optic atrophy. Nat Genet 2000; 26: 207–210. 11017079 10.1038/79936

[bib10] 10Waterham HR, Koster J, van Roermund CW, Mooyer PA, Wanders RJ, Leonard JV et al. A lethal defect of mitochondrial and peroxisomal fission. N Engl J Med 2007; 356: 1736–1741. 17460227 10.1056/NEJMoa064436

[bib11] 11Züchner S, Mersiyanova IV, Muglia M, Bissar-Tadmouri N, Rochelle J, Dadali EL et al. Mutations in the mitochondrial GTPase mitofusin 2 cause Charcot-Marie-Tooth neuropathy type 2 A. Nat Genet 2004; 36: 449–451. 15064763 10.1038/ng1341

[bib12] 12Lokireddy S, Wijesoma IW, Bonala S, Wei M, Sze SK, McFarlane C et al. Myostatin is a novel tumoral factor that induces cancer cachexia. Biochem J 2012; 446: 23–36. 22621320 10.1042/BJ20112024 PMC3408049

[bib13] 13Jheng HF, Tsai PJ, Guo SM, Kuo LH, Chang CS, Su IJ et al. Mitochondrial fission contributes to mitochondrial dysfunction and insulin resistance in skeletal muscle. Mol Cell Biol 2011; 32: 309–319. 22083962 10.1128/MCB.05603-11 PMC3255771

[bib14] 14Liu R, Jin P, Liqun Yu, Wang Y, Han L, Shi T et al. Impaired mitochondrial dynamics and bioenergetics in diabetic skeletal muscle. PLoS One 2014; 9: e92810. 24658162 10.1371/journal.pone.0092810 PMC3962456

[bib15] 15Eisner V, Lenaers G, Hajnóczky G. Mitochondrial fusion is frequent in skeletal muscle and supports excitation-contraction coupling. J Cell Biol 2014; 205: 179–195. 24751540 10.1083/jcb.201312066 PMC4003250

[bib16] 16Romanello V, Guadagnin E, Gomes L, Roder I, Sandri C, Petersen Y et al. Mitochondrial fission and remodelling contributes to muscle atrophy. EMBO J 2010; 29: 1774–1785. 20400940 10.1038/emboj.2010.60 PMC2876965

[bib17] 17Kim B, Kim JS, Yoon Y, Santiago MC, Brown MD, Park JY et al. Inhibition of Drp1-dependent mitochondrial division impairs myogenic differentiation. Am J Physiol 2013; 305: R927–R938. 10.1152/ajpregu.00502.2012 23904108

[bib18] 18Kanisicak O, Mendez JJ, Yamamoto S, Yamamoto M, Goldhamer DJ. Progenitors of skeletal muscle satellite cells express the muscle determination gene, MyoD. Dev Biol 2009; 332: 131–141. 19464281 10.1016/j.ydbio.2009.05.554 PMC2728477

[bib19] 19Reeds PJ, Fiorotto ML. Growth in perspective. Proc Nutr Soc 2007; 49: 411–420. 10.1079/pns19900048 2080173

[bib20] 20Irwin WA, Bergamin N, Sabatelli P, Reggiani C, Megighian A, Merlini L et al. Mitochondrial dysfunction and apoptosis in myopathic mice with collagen VI deficiency. Nat Genet 2003; 35: 367–371. 14625552 10.1038/ng1270

[bib21] 21Martinus RD, Garth GP, Webster TL, Cartwright P, Naylor DJ, Høj PB et al. Selective induction of mitochondrial chaperones in response to loss of the mitochondrial genome. Eur J Biochem 1996; 240: 98–103. 8797841 10.1111/j.1432-1033.1996.0098h.x

[bib22] 22Horibe T, Hoogenraad NJ. The chop gene contains an element for the positive regulation of the mitochondrial unfolded protein response. PLoS One 2007; 2: e835. 17848986 10.1371/journal.pone.0000835 PMC1950685

[bib23] 23Chen WW, Birsoy K, Mihaylova MM, Snitkin H, Stasinski I, Yucel B et al. Inhibition of ATPIF1 ameliorates severe mitochondrial respiratory chain dysfunction in mammalian cells. Cell Rep 2014; 7: 27–34. 24685140 10.1016/j.celrep.2014.02.046 PMC4040975

[bib24] 24Wek RC, Jiang HY, Anthony TG. Coping with stress: eIF2 kinases and translational control. Biochem Soc Trans 2006; 34: 7–11. 16246168 10.1042/BST20060007

[bib25] 25Rath E, Berger E, Messlik A, Nunes T, Liu B, Kim SC et al. Induction of dsRNA-activated protein kinase links mitochondrial unfolded protein response to the pathogenesis of intestinal inflammation. Gut 2012; 61: 1269–1278. 21997551 10.1136/gutjnl-2011-300767 PMC4514769

[bib26] 26Palam LR, Baird TD, Wek RC. Phosphorylation of eIF2 facilitates ribosomal bypass of an inhibitory upstream ORF to enhance CHOP translation. J Biol Chem 2011; 286: 10939–10949. 21285359 10.1074/jbc.M110.216093 PMC3064149

[bib27] 27Ebert SM, Monteys AM, Fox DK, Bongers KS, Shields BE, Malmberg SE et al. The transcription factor ATF4 promotes skeletal myofiber atrophy during fasting. Mol Endocrinol 2010; 24: 790–799. 20197309 10.1210/me.2009-0345 PMC2852358

[bib28] 28Suomalainen A, Elo JM, Pietiläinen KH, Hakonen AH, Sevastianova K, Korpela M et al. FGF-21 as a biomarker for muscle-manifesting mitochondrial respiratory chain deficiencies: a diagnostic study. Lancet Neurol 2011; 10: 806–818. 21820356 10.1016/S1474-4422(11)70155-7 PMC7568343

[bib29] 29Wu S., Grunwald T, Kharitonenkov A, Dam J, Jockers R, De Luca F. et al. Increased expression of fibroblast growth factor 21 (FGF21) during chronic undernutrition causes growth hormone insensitivity in chondrocytes by inducing leptin receptor overlapping transcript (LEPROT) and leptin receptor overlapping transcript-like 1 (LEPROTL1) expression. J Biol Chem 2013; 288: 27375–27383. 23940039 10.1074/jbc.M113.462218 PMC3779732

[bib30] 30Ebert SM, Dyle MC, Kunkel SD, Bullard SA, Bongers KS, Fox DK et al. Stress-induced skeletal muscle Gadd45a expression reprograms myonuclei and causes muscle atrophy. J Biol Chem 2012; 287: 27290–27301. 22692209 10.1074/jbc.M112.374777 PMC3431665

[bib31] 31Inagaki T, Dyle MC, Kunkel SD, Bullard SA, Bongers KS, Fox DK et al. Inhibition of growth hormone signaling by the fasting-induced hormone FGF21. Cell Metab 2008; 8: 77–83. 18585098 10.1016/j.cmet.2008.05.006 PMC2575072

[bib32] 32Touvier T, Conte-Auriol F, Briand O, Cudejko C, Paumelle R, Caron S et al. LEPROT and LEPROTL1 cooperatively decrease hepatic growth hormone action in mice. J Clin Invest 2009; 119: 3830–3838. 19907080 10.1172/JCI34997 PMC2786784

[bib33] 33Ogata T, Yamasaki Y. Ultra-high-resolution scanning electron microscopy of mitochondria and sarcoplasmic reticulum arrangement in human red, white, and intermediate muscle fibers. Anat Rec 1997; 248: 214–223. 9185987 10.1002/(SICI)1097-0185(199706)248:2<214::AID-AR8>3.0.CO;2-S

[bib34] 34Kasahara A, Cipolat S, Chen Y, Dorn GW, Scorrano L. Mitochondrial fusion directs cardiomyocyte differentiation via calcineurin and Notch signaling. Science 2013; 342: 734–737. 24091702 10.1126/science.1241359

[bib35] 35Frieden M, James D, Castelbou C, Danckaert A, Martinou JC, Demaurex N et al. Ca2+ Homeostasis during mitochondrial fragmentation and perinuclear clustering induced by hFis1. J Biol Chem 2004; 279: 22704–22714. 15024001 10.1074/jbc.M312366200

[bib36] 36Al-Mehdi AB, Pastukh VM, Swiger BM, Reed DJ, Patel MR et al. Perinuclear mitochondrial clustering creates an oxidant-rich nuclear domain required for hypoxia-induced transcription. Sci Signal 2012; 5: ra47. 22763339 10.1126/scisignal.2002712 PMC3565837

[bib37] 37Elachouri G, Vidoni S, Zanna C, Pattyn A, Boukhaddaoui H, Gaget K et al. OPA1 links human mitochondrial genome maintenance to mtDNA replication and distribution. Genome Res 2011; 21: 12–20. 20974897 10.1101/gr.108696.110 PMC3012919

[bib38] 38Alter J, Bengal E. Stress-induced C/EBP homology protein (CHOP) represses MyoD transcription to delay myoblast differentiation. PLoS One 2011; 6: e29498. 22242125 10.1371/journal.pone.0029498 PMC3248460

[bib39] 39Muñoz JP, Zorzano A. Mfn2 modulates the unfolded protein response. Cell Cycle 2014; 13: 0–1. 10.4161/cc.27782 24419149

[bib40] 40Ngoh GA, Papanicolaou KN, Walsh K. Loss of Mitofusin 2 promotes endoplasmic reticulum stress. J Biol Chem 2012; 287: 20321–20332. 22511781 10.1074/jbc.M112.359174 PMC3370214

[bib41] 41Eley HL, Russell ST, Tisdale MJ. Attenuation of muscle atrophy in a murine model of cachexia by inhibition of the dsRNA-dependent protein kinase. Br J Cancer 2007; 96: 1216–1222. 17387345 10.1038/sj.bjc.6603704 PMC2360141

[bib42] 42Eley HL, Tisdale MJ. Skeletal muscle atrophy, a link between depression of protein synthesis and increase in degradation. J Biol Chem 2007; 282: 7087–7097. 17213191 10.1074/jbc.M610378200

[bib43] 43Russell ST, Rajani S, Dhadda RS, Tisdale MJ. Mechanism of induction of muscle protein loss by hyperglycaemia. Exp Cell Res 2009; 315: 16–25. 18973755 10.1016/j.yexcr.2008.10.002

[bib44] 44Tyynismaa H, Carroll CJ, Raimundo N, Ahola-Erkkilä S, Wenz T, Ruhanen H et al. Mitochondrial myopathy induces a starvation-like response. Hum Mol Genet 2010; 19: 3948–3958. 20656789 10.1093/hmg/ddq310

[bib45] 45Kopchick JJ, Laron Z. Is the Laron mouse an accurate model of Laron syndrome? Mol Genet Metab 1999; 68: 232–236. 10527674 10.1006/mgme.1999.2890

[bib46] 46Badaloni A, Bonanomi D, Albieri I, Givogri I, Bongarzone E, Valtorta F et al. Transgenic mice expressing a dual, CRE-inducible reporter for the analysis of axon guidance and synaptogenesis. Genesis 2007; 45: 405–412. 17554764 10.1002/dvg.20307

[bib47] 47Rigamonti E, Touvier T, Clementi E, Manfredi AA, Brunelli S, Rovere-Querini P et al. Requirement of inducible nitric oxide synthase for skeletal muscle regeneration after acute damage. J Immunol 2013; 190: 1767–1777. 23335752 10.4049/jimmunol.1202903 PMC3566578

[bib48] 48D'Antonio M, Musner N, Scapin C, Ungaro D, Del Carro U, Ron D et al. Resetting translational homeostasis restores myelination in Charcot-Marie-Tooth disease type 1B mice. J Exp Med 2013; 210: 821–838. 23547100 10.1084/jem.20122005 PMC3620355

[bib49] 49Frezza C, Cipolat S, Scorrano L. Organelle isolation: functional mitochondria from mouse liver, muscle and cultured fibroblasts. Nat Prot 2007; 2: 287–295. 10.1038/nprot.2006.478 17406588

[bib50] 50Taillandier D, Aurousseau E, Combaret L, Guezennec CY, Attaix D. Regulation of proteolysis during reloading of the unweighted soleus muscle. Int J Biochem Cell Biol 2003; 35: 665–675. 12672458 10.1016/s1357-2725(03)00004-9

