# Supplementary material for: Muscle-specific Drp1 overexpression impairs skeletal muscle growth via translational attenuation
Source: Cell Death Dis. 2015 Feb 26;6(2):e1663–. doi: 10.1038/cddis.2014.595 (PMC4669802; doi:10.1038/cddis.2014.595)
Supplement: Supplementary Table S1 [file cddis2014595x1.doc]

| **Table S1. Metabolic parameters in P100 wild-type and Drp/MC mice** | | |
| --- | --- | --- |
|  | WT | Drp/MC |
| Food intake (g/day) | 3,8 ± 0,2 | 3,7 ± 0,2 |
| Food intake (g/day/BW) | 0,121 ± 0,007 | 0,150 ± 0,007 * |
| Body temperature (°C) | 38,1 ± 0,1 | 37,9 ± 0,2 |
| Fed glycemia (mg/dL) | 160 ± 32 | 157 ± 21 |
| Fasted glycemia (mg/dL) | 77 ± 13 | 69 ± 11 |
| Insulin (pM) | 193 ± 34 | 164 ± 33 |
| Glucagon (pM) | 12 ± 1 | 12 ± 1 |
| Triglycerides (mg/dL) | 137 ± 20 | 123 ± 8 |
| Leptin (pM) | 112 ± 16 | 136 ± 19 |
| Data are given +- SEM. N≥5 mice per group. T test vs WT, *p<0.05 | | |

| **Table S2. Morphological parameters measured by computerized tomography in P100 wild-type and Drp/MC male mice** | | |
| --- | --- | --- |
|  | WT | Drp/MC |
| Body length (mm) | 168,5 ± 2,4 | 167,0 ± 1,3 |
| Tibial length (mm) | 1,528 ± 0,053 | 1,594 ± 0,009 |
| Tibial width (mm) | 0,215 ± 0,008 | 0,188 ± 0,003 ** |
| Body volume (cm^3^) | 27,88 ± 0,73 | 21,71 ± 0,59 *** |
| Muscle volume (cm^3^) | 12,51 ± 0,11 | 9,07 ± 0,25 *** |
| Adipose volume (cm^3^) | 7,53 ± 0,49 | 5,72 ± 0,28 ** |
| Skeleton volume (cm^3^) | 3,09 ± 0,05 | 2,55 ± 0,08 *** |
| Muscle / body volume | 45,05 ± 0,90 | 41,86 ± 0,98 * |
| Adipose / body volume | 26,85 ± 1,05 | 26,36 ± 1,15 |
| Skeleton / body volume | 11,13 ± 0,20 | 11,74 ± 0,15 * |
| Data are given +- SEM. N=8 mice per group. T test vs WT, *p<0.05 **p<0.01 ***p<0.001 | | |

| **Table S3. List of antibodies used for IF, WB and IP experiments** | | |
| --- | --- | --- |
| **Antibody Name** | **Catalog No.** | **Company Name** |
| **4EBP1** | 9644 | Cell signaling |
| **P-4EBP1** | 2855 | Cell signaling |
| **Actin** | sc-1616 | Santa Cruz Biotechnology |
| **Akt** | 9272 | Cell signaling |
| **P-Akt** | 9271 | Cell Signaling |
| **AMPKa** | 5832 | Cell Signaling |
| **P-AMPKa** | 2531 | Cell Signaling |
| **ATPIF1** | ab110277 | Abcam |
| **ClpP** | WH008192M1 | Sigma-Aldrich |
| **Complex II** | A11142 | Molecolar Probes |
| **Cyclophilin D** | MS04 | Mitosciences |
| **Drp1** | D80320 | BD transduction |
| **eIF2alpha** | 9722 | Cell Signaling |
| **P-eIF2alpha** | 9721 | Cell Signaling |
| **GAPDH** | sc-25778 | Santa Cruz biotechnology |
| **GSK3β** | 9315 | Cell Signaling |
| **P-GSK3β** | 9323 | Cell Signaling |
| **Hsp60** | sc-1052 | Santa Cruz biotechnology |
| **Laminin** | L9393 | Sigma-Aldrich |
| **LC3** | L7543 | Sigma-Aldrich |
| **Mfn1** | H00055669-M04 | Abnova |
| **Mfn2** | H00009927-M03 | Abnova |
| **MHC** | MF20 | Developmental Studies  Hybridoma Bank |
| **MHC2a** | SC-71 | ATCC |
| **MHC2b** | BF-F3 | ATCC |
| **Myc** | C3956 | Sigma-Aldrich |
| **Opa1** | 612606 | BD transduction |
| **Pax7** |  | Developmental Studies  Hybridoma Bank |
| **PKR** | sc-708 | Santa Cruz biotechnology |
| **P-PKR** | ab32036 | Abcam |
| **S6** | 2317 | Cell Signaling |
| **P-S6** | 2215 | Cell Signaling |
| **Sv2** |  | Developmental Studies  Hybridoma Bank |
| **Tim23** | 611222 | BD transduction |
| **b-Tubulin** | MMS-411R | Covance |
| **Vinculin** | V9264 | Sigma |
|  | | |

| **Table S4. List of primers** | | |
| --- | --- | --- |
| **Gene** | **Primers sequences** | **Product size (bp)** |
| **28S** | Forward: 5’- AAACTCTGGTGGAGGTCCGT -3’  Reverse: 5’- CTTACCAAAAGTGGCCCACTA -3’ | 296 |
| **36B4** | Forward: 5’- AGATTCGGGATATGCTGTTGG 3’  Reverse: 5’- AAAGCCTGGAAGAAGGAGGTC -3’ | 132 |
| **Asns** | Forward: 5’- ATTACGACAGTTCGGGCATC -3’  Reverse: 5’- TCTCAGTTCGAGACCGTGTG -3’ | 245 |
| **Atf3** | Forward: 5’- GACCCCTGGAGATGTCAGTC -3’  Reverse: 5’- TCTGACTCTTTCTGCAGGCA-3’ | 147 |
| **Atf4** | Forward: 5’- AGCAAAACAAGACAGCAGCC -3’  Reverse: 5’- ACTCTCTTCTTCCCCCTTGC -3’ | 192 |
| **Atf6** | Forward: 5’-GAACTTCGAGGCTGGGTTCA -3’  Reverse: 5’- TCCAGGGGAGGCGTAATACA -3’ | 203 |
| **Atp5o** | Forward: 5’- TCTGGCGCCAGTAGTCTCTT -3’  Reverse: 5’- AGATGATACCCTGGGTGTTG -3’ | 431 |
| **Atpif1** | Forward: 5’- GGTGTCTGGGGTATGAAGGTC -3’  Reverse: 5’- CCTTTTCTCGTTTTCCGAAGGC -3’ | 121 |
| **Cish** | Forward: 5’- AGTACGCCGATTCTAGCTTC -3’  Reverse: 5’- CTGTCGCTCCGGGTGTCAG -3’ | 129 |
| **COII** | Forward: 5’- AACCATAGGGCACCAATGATAC -3’  Reverse: 5’- GGATGGCATCAGTTTTAAGTCC -3’ | 232 |
| **Cox5b** | Forward: 5’- AGGCAGCTTCAGGCACCAAG -3’  Reverse: 5’- GGTGGGGCACCAGCTTGTAA -3’ | 173 |
| **Cytb** | Forward: 5’- ACGCCATTCTACGCTCTATC -3’  Reverse: 5’- GCTTCGTTGCTTTGAGGTGT -3’ | 109 |
| **Cycs** | Forward: 5’- GGAGGCAAGCATAAGACTGG -3’  Reverse: 5’- TCCATCAGGGTATCCTCTCC -3’ | 130 |
| **Ddit3/chop** | Forward: 5’- ATATCTCATCCCCAGGAAACG -3’  Reverse: 5’- TCTTCCTTGCTCTTCCTCCTC -3’ | 187 |
| **Fbxo32/**  **ATROGIN1** | Forward: 5’- TTCAGCAGCCTGAACTACGA-3’  Reverse: 5’- GGCAGTCGAGAAGTCCAGTC-3’ | 182 |
| **Fgf21** | Forward: 5’- CTGGGGGTCTACCAAGCATA -3’  Reverse: 5’- CACCCAGGATTTGAATGACC -3’ | 219 |
| **Gadd45a** | Forward: 5’- CGGTGATGGCATCCGAATGGAAAT-3’  Reverse: 5’- TCTGCAAAGTCATCTCTGAGCCCT -3’ | 165 |
| **Ghr** | Forward: 5’- CGATTCACCAAGTGTCGTTC -3’  Reverse: 5’- TCAGGGCATTCTTTCCATTC -3’ | 178 |
| **Hspa5/**  **Grp78** | Forward: 5’- TGTGGTACCCACCAAGAAGTC -3’  Reverse: 5’- TTCAGCTGTCACTCGGAGAAT -3’ | 219 |
| **Grp94** | Forward: 5’- CTCAGAAGACGCAGAAGACTCA -3’  Reverse: 5’- AAAACTTCACATTCCCTCTCCA -3’ | 159 |
| **Igf1** | Forward: 5’- GTGTGGACCGAGGGGCTTTTACTTC -3’  Reverse: 5’- GCTTCAGTGGGGCACAGTACATCTC -3’ | 145 |
| **Jak2** | Forward: 5’- GATGGCGGTGTTAGACATGA -3’  Reverse: 5’- TGCTGAATGAATCTGCGAAA -3’ | 93 |
| **Leprot** | Forward: 5’- GGGCTGACTTTTCTTATGCTG -3’  Reverse: 5’- CCCAGTGGTGAAGAAATACGC -3’ | 179 |
| **Leprotl1** | Forward: 5’- GCCCTTCCGATATACAACCA -3’  Reverse: 5’- CTCCTTACACGCGTTGC -3’ | 128 |
| **Mthfd2** | Forward: 5’- CATGGGGCGTGTGGGAGATAAT -3’  Reverse: 5’- CCGGGCCGTTCGTGAGC -3’ | 138 |
| **Mul1** | Forward: 5’- AGGGCATTCTTTCAGAAGCA -3’  Reverse: 5’- GGGGTGGAACTTCTCGTACA -3’ | 328 |
| **ND1** | Forward: 5’- GTTGGTCCATACGGCATTTT -3’  Reverse: 5’- TGGGTGTGGTATTGGTAGGG -3’ | 164 |
| **ND2** | Forward: 5’- CCTATCACCCTTGCCATCAT -3’  Reverse: 5’- GAGGCTGTTGCTTGTGTGAC-3’ | 193 |
| **Ndufb2** | Forward: 5’- GGTGATCCAGGGTGAGTTCT -3’  Reverse: 5’- TGTCAGTCCTCGTCATCAGG -3’ | 158 |
| **P21** | Forward: 5’- CAAAGTGTGCCGTTGTCTCT -3’  Reverse: 5’- GTCAAAGTTCCACCGTTCTC -3’ | 111 |
| **Pecam** | Forward: 5’- ATGGAAAGCCTGCCATCATG -3’  Reverse: 5’- TCCTTGTTGTTCAGCATCAC -3’ | 235 |
| **Ppia** | Forward: 5’-CATACGGGTCCTGGCATCTTGTCC -3’  Reverse: 5’-TGGTGATCTTCTTGCTGGTCTTGC -3’ | 198 |
| **Sdhb** | Forward: 5’- GGACCTATGGTGTTGGATGC -3’  Reverse: 5’- GTGTGCACGCCAGAGTATTG -3’ | 138 |
| **Slc3a2** | Forward: 5’- GGGGAGCGTACTGAATCCCT-3’  Reverse: 5’- CTGAAGGCCAAGCTCATCCC -3’ | 191 |
| **Slc6a9** | Forward: 5’- GTTGGCGCTTTGTTTCTCCG -3’  Reverse: 5’- TCTGCTTGGCTTTGTGGCAT -3’ | 232 |
| **Trim63/**  **MuRF1** | Forward: 5’- ACCTGCTGGTGGAAAACATC-3’  Reverse: 5’- CTTCGTGTTCCTTGCACATC -3’ | 95 |
|  | | |

Abbreviations are: 28S, 28S ribosomal RNA; 36B4, ribosomal protein, large, P0 (Rplp0); Asns, asparagine synthetase; Atf3, activating transcription factor 3; Atf4, activating transcription factor 4; Atf6, activating transcription factor 6; ATP5o, ATP synthase, H+ transporting, mitochondrial F1 complex, O subunit; Atpif1, ATPase inhibitory factor 1; Cish, cytokine inducible SH2-containing protein; COII, cytochrome c oxidase subunit II; Cytb, cytochrome b; Cycs, cytochrome c, somatic; Ddit3, DNA-damage inducible transcript 3; Fbxo32, F-box protein 32; Fgf21, fibroblast growth factor 21; Gadd45a, growth arrest and DNA-damage-inducible 45 alpha; Ghr, growth hormone receptor; Hspa5, heat shock protein 5; Grp94, heat shock protein 90, beta; Igf1, insulin-like growth factor 1; Jak2, Janus kinase 2; Leprot, leptin receptor overlapping transcript; Leprotl1, leptin receptor overlapping transcript-like 1; Mthfd2, methylenetetrahydrofolate dehydrogenase (NAD+ dependent), methenyltetrahydrofolate cyclohydrolase; Mul1, mitochondrial ubiquitin ligase activator of NFKB 1; ND1 or 2, NADH dehydrogenase subunit 1 or 2; P21, cyclin-dependent kinase inhibitor 1A; Ppia, peptidylprolyl isomerase A; Sdhb, succinate dehydrogenase complex, subunit B, iron sulfur; Slc3a2, solute carrier family 3 (activators of dibasic and neutral amino acid transport), member 2; Slc6a9, solute carrier family 6 (neurotransmitter transporter, glycine), member 9; Trim63, tripartite motif-containing 63.
