# Supplementary material for: Muscle-specific Drp1 overexpression impairs skeletal muscle growth via translational attenuation
Source: Cell Death Dis. 2015 Feb 26;6(2):e1663–. doi: 10.1038/cddis.2014.595 (PMC4669802; doi:10.1038/cddis.2014.595)
Supplement: Supplementary Information 1 [file cddis2014595x2.doc]

**Muscle-specific Drp1 overexpression impairs skeletal muscle growth via translational attenuation**

Thierry Touvier^1^, Clara De Palma^2^, Elena Rigamonti^3^, Alessandra Scagliola^3,4^, Elena Incerti^3,4^, Laetitia Mazelin^5^, Jean-Luc Thomas^5^, Maurizio D'Antonio^6^, Letterio Politi^7^, Laurent Schaeffer^5^, Emilio Clementi^1,2*^ & Silvia Brunelli^3,4*^.

**SUPPLEMENTARY METHODS AND PROTOCOLS**

**Generation of skeletal muscle-specific Drp1 transgenic mice and animal experimentation.**

To generate the Drp1 transgenic mice, a cDNA containing the human Drp1 coding region (pCMV6-XL4-DNM1L, Origene) was first amplified by PCR with primers containing restrictions sites and a Myc sequence in 3' and then subcloned into pIRES-EGFP vector (Clontech). Drp1-IresEGFP sequence was then excised and inserted into the piGAP vector ^1^. piGAP-Drp1 contains a chicken β-actin promoter fused to the cytomegalovirus enhancer, a nuclear DsRed and the rabbit β-globin polyadenylation sequence flanked at both ends by loxP sequences and the Drp1-Ires-Egfp sequence. Upon CRE-mediated recombination of the loxP sites, the DsRed-polyA sequence is removed, permitting transcription and dicistronic translation of Drp1-Ires-Egfp. The entire piGAP-Drp1 transcription unit was then excised with the restriction enzyme SalI, gel purified and injected into fertilized oocytes of FVB/N females (Core Facility for Conditional Mutagenesis, San Raffaele Scientific Institute, Milan, Italy). Transgenic mice were identified by PCR analysis of tail DNA using the following primers: TgDrp1 forward: 5′- GCATTACAAGGAGCCAGTCAA -3′; and TgDrp1 reverse: 5′- CACCCTCAAAGGCATCACC -3′. Drp transgenic mice were then crossed with heterozygous MyoD^iCre^ knockin mice, which are phenotypically wild type ^2^. FGF21 plasma levels were measured using an ELISA kit (MF2100, R&D Systems), and plasma glucose concentrations were determined using Glucotrend 2 (Roche Diagnostics) on tail bleeds in fed animals or after a 6-hour fasting.

**Growth hormone response**

Mice were administered saline or hGH (1,5mg/Kg) with an intra-peritoneal injection. Mice were sacrificed after 4 hours and livers and quadriceps were immediately frozen for subsequent analysis.

**Analysis of muscle functional parameters**

Mice were familiarized on the treadmill by running for 10 min every other day at 15 cm/sec for 1 week before beginning the exercise training protocol. For the power test, mice were initially placed on a +5 degree inclined treadmill at 15cm/sec; the speed was then increased by 2cm/sec every minute until exhaustion. For the resistance test, mice were placed into a +5° inclined treadmill at 15cm/sec; the speed was then increased by 3cm/sec every 12 minutes until exhaustion.

**Single fibre preparation**

Gastrocnemius muscles of 2-mo-old mice were dissected and digested in 0.1% collagenase type V (Sigma-Aldrich). Individual myofibres were dissociated. For immunofluorescence studies myofibres were fixed for 10 min in 4% PFA/PBS (Sigma-Aldrich) and subsequently washed with PBS and processed for IF as in ^3^.

**In vivo electroporation**

10-week-old male mice were anaesthetised with an intra-peritoneal injection of a ketamine (100mg/Kg) / xylazine (10mg/Kg) mixture. TA muscles were injected with 30ul of 0.45% NaCl containing 20ug of DNA (pDsRed2-mito, pLC3-YFP) and subsequently electroporated with 1 cm^2^ plaque electrodes placed on each side of the leg and eight 200 V/cm pulses of 20 ms applied at 2 Hz (BTX ECM 830 electroporator). Muscles were collected seven days after the electroporation.

**Oxygen consumption on permeabilised fibres**

A portion of freshly isolated TA muscle was placed into ice-cold relaxing solution BIOPS containing 10 mM CaK2-EGTA, 7.23 mM K2-EGTA, 20 mM imidazole, 20 mM taurine, 50 mM K-MES, 0.5 mM dithiothreitol, 6.56 mM MgCl2, 5.77 mM ATP and 15 mM phosphocreatine adjusted to pH 7.1. The fibres were kept at 4°C until further preparation.

TA muscle was carefully dissected free of any fat and connective tissue and separated into bundles under a dissection microscope with fine forceps. Fibres were then collected, immersed in fresh BIOPS solution supplemented with saponin (50 μg/ml) and incubated at 4°C on a rotator for 30 min. Bundles were then washed twice in respiration buffer MIRO5 (0.5 mM EGTA, 3 mM MgCl2, 60 mM K-lactobionate, 20 mM taurine, 10 mM KH2PO4, 20 mM HEPES, 110 mM sucrose, and 1 g/L BSA (fatty acid free), pH 7.1) for 5 minutes on a rotator at 4°C to remove any residual permeabilisation solution. Permeabilised fibre bundles were blotted dry, weighed (1-2 mg) and transferred to a high-resolution respirometer (Oroboros Instruments) containing 2 ml of MiR06 (MiR05 supplemented with 280 U/ml catalase at 37.0°C). Oxygen concentration (mM), and oxygen flux per muscle mass (pmol O_2_*s^-1^*mg^-1^Ww) were recorded online using DatLab software (Oroboros Instruments).

After calibration of the oxygen sensors at air saturation, a few µl of H_2_O_2_ was introduced to reach O_2_ concentration of 400 µM.

The electron flow through flow through mitochondrial complexes I and II was measured followed titrations of all of substrates, uncouplers and inhibitors (SUIT) as previously described ^4^. The measurement of complex IV respiration was obtained by addition of the TMPD (N,N,N’,N’-tetramethyl-p-phenylenediamine dihydrochloride) and ascorbate ^5^. Oxygen fluxes were corrected by subtracting residual oxygen consumption from each measured mitochondrial steady-state.

**Computerised tomography**

Computerised tomography (CT) scans were performed on a human-grade 64-channel multislice apparatus (Light Speed VCT; GE Healthcare). The imaging protocol included a biplanar scout and a helical volumetric CT acquisition with coverage of the whole body, with a tube speed rotation of 0.5 seconds (s), 0.625-mm slice thickness and 0.3-mm/s table motion, 120 kV, 200 mA, reconstruction field of view of 17 cm, and matrix of 512x512. CT images were filtered with both the standard parenchyma and high-resolution bone algorithms. On a dedicated workstation (Advantage 4.4; GE Healthcare) the total body, skeletal, fat and muscle volumes (in cubic centimetres) of each mouse were measured after applying an automatic segmentation (bone threshold >160 Hounsfield Unit; density range from -190 to -10 H.U. for fat; density range from 10 to 65 H.U. for muscle). Femur length was determined by measuring its long axis between the two epiphyses, and its width was measured in the middle of the diaphysis. The mouse total body length was measured using curved reformatted images.

**Acute muscle damage**

WT and Drp/MC mice were anesthetized and subsequently injected with cardiotoxin in the TA muscle (CTX; Naja mossambica mossambica, Sigma-Aldrich; 50 ul, 15 uM for TA muscles). Mice were sacrificed at 3, 5, 7, 10, and 14 days after injury. Muscles were then collected for histological analysis.

**Histology and Immunofluorescence**

For histology, muscles were collected and directly frozen in liquid nitrogen cool isopentane to allow preparation of 10 micron thick sections for both morphological and immunofluorescence analysis. Serial muscle sections were stained with H&E (Sigma-Aldrich) or succinic dehydrogenase staining (Bio-Optica). For immunofluorescence, sections were fixed with 4% paraformaldehyde and then blocked and permeabilised with 5% BSA and 0.1% Triton in PBS before incubation with primary antibody (list is provided below). Appropriate Alexa Fluor (Alexa 488 or Alexa 594)-conjugated Abs (1:500; Invitrogen) were used as second-step reagents. Specimens were counterstained with Hoechst 33342 (Molecular Probes) and analysed using a Nikon Eclipse 55i microscope (Nikon). CSA and central nucleation analyses were carried out on 500–750 fibres per muscle on muscle after immunostaining for laminin or staining with H&E, using Image J software (<http://rsbweb.nih.gov/ij/>). For analysis of muscle fibre types, TA muscle sections were immunostained with mouse IgG1 anti-MHC2A (SC-71) or mouse IgM anti-MHC2B (BF-F3).

**Measurement of ATP formation.**

Mitochondria were obtained as described ^6^ with slight modifications. Fibres were dissected, trimmed and cleaned of visible fat and connective tissue, and digested in ATP medium, containing 50 mM Tris-HCl (pH 7.4), 100 mM KCl, 5 mM MgCl_2_, 1.8 mM ATP, 1 mM EDTA, and 0.1% collagenase type V for 10 min at 37°C under strong agitation. After centrifugation, the tissues were homogenized with Ultra-Turrax T10 (IKA) in ATP medium. The mitochondrial fraction was obtained by different centrifugations (380g and 10000g for 5 min at 4°C) and then resuspended in mitochondria resuspension buffer, containing 12.5 mM Tris acetate, 225 mM sucrose, 44 mM KH_2_PO_4_ and 6 mM EDTA. OXPHOS-ATP in isolated mitochondria was measured by luciferin-luciferase method as described ^7^. Briefly, mitochondria were plated in 96 wells and treated with buffer (pH 7.4) containing 150mM KCl, 25 mM Tris-HCl, 2 mM EDTA, 0.1% BSA, 10 mM KH_2_PO_4_ and 0.1 mM MgCl_2_, 0.8 M malate, 2 M glutamate, 500 mM ADP, 100 mM luciferin, and 1 mg/ml luciferase. Oligomycin (2 µg/ml) was also added to detect the presence of glycolytic ATP in our samples. OXPHOS-ATP was measured using a GloMax luminometer (Promega).

**REFERENCE TO EXTENDED METHODS AND PROTOCOLS**

1. Badaloni, A. *et al.* Transgenic mice expressing a dual, CRE-inducible reporter for the analysis of axon guidance and synaptogenesis. *Genesis* **45,** 405–412 (2007).

2. Kanisicak, O., Mendez, J. J., Yamamoto, S., Yamamoto, M. & Goldhamer, D. J. Progenitors of skeletal muscle satellite cells express the muscle determination gene, MyoD. *Dev Biol* **332,** 131–141 (2009).

3. Deponti, D. *et al.* Necdin mediates skeletal muscle regeneration by promoting myoblast survival and differentiation. *J Cell Biol* **179,** 305–319 (2007).

4. Votion, D.-M., Gnaiger, E., Lemieux, H., Mouithys-Mickalad, A. & Serteyn, D. Physical fitness and mitochondrial respiratory capacity in horse skeletal muscle. *PLoS ONE* **7,** e34890 (2012).

5. Kuznetsov, A. V. *et al.* Analysis of mitochondrial function in situ in permeabilized muscle fibers, tissues and cells. *Nature protocols* **3,** 965–976 (2008).

6. Frezza, C., Cipolat, S. & Scorrano, L. Organelle isolation: functional mitochondria from mouse liver, muscle and cultured fibroblasts. *Nature protocols* **2,** 287–295 (2007).

7. De Palma, C. *et al.* Nitric oxide inhibition of Drp1-mediated mitochondrial fission is critical for myogenic differentiation. *Cell Death Differ* **17,** 1684–1696 (2010).

**LEGENDS TO EXPANDED VIEW FIGURES**

**Figure S1.** Neuromuscular junction is not altered in Drp/MC muscle.

TA muscle from P100 WT and transgenic animals was fixed and muscle fibres were isolated and stained with fluorescent α-bungarotoxin and immunostained with Sv2 specific antibody. Five representative neuromuscular junctions for each genotype are presented.

**Figure S2.** Fibre typology is unaltered in Drp/MC muscle.

Transversal sections of TA muscle were immunostained with MHC2A (green) and MHC2B (red) specific antibodies. Original magnification x20. Scale bar 50um.

**Figure S3.** Regeneration kinetic after muscle injury is not altered in Drp/MC mice.

(A) Representative images of TA muscle cross-sections from P100 WT and Drp/MC mice at 3, 5, 7, and 14 days post-cardiotoxin (CTX) injury, stained for H&E, original magnification x10. Scale bar 50um. (B) Quantification of the number of centrally nucleated myofibres per um^2^ in WT and Drp/MC regenerating muscles 5 days after injury (n=2 mice per genotype). (C,D) Frequency histogram showing the distribution of myofibres CSA in the TA muscles from WT and Drp/MC mice at 7 (C) and 14 (D) days postinjury. Average CSA of myofibres from regenerating WT and Drp/MC TA muscles at 7 (C) and 14 (D) days post injury. Data pooled from 750 fibres from n=2 mice per genotype. Error bars represent SEM.

**Figure S4.** Ubiquitin-proteasome and autophagy systems are not deregulated in growing and adult Drp/MC muscles.

(A) RT-qPCR analysis of atrophy-related genes Atrogin-1, MuRF1 and Mul-1 mRNA levels in quadriceps from P1, P7, P25 and P100 WT and Drp/MC mice (n=4 per genotype). (B) Western blot analysis of LC3 and p62 protein expression levels in quadriceps from P7, P25 and P100 WT and Drp/MC mice. (C) Western blot analysis of phosphorylated and total AMPK expression levels in quadriceps from adult WT and Drp/MC mice (n=4 per genotype). Error bars represent SEM.

**Figure S5.** COX staining of representative transversal sections from P100 WT and Drp/MC TA muscle. Original magnification x20. Scale bar 50um.

**Figure S6.** Mitophagy is not activated in Drp/MC TA muscle.

(A) Western blot analysis of PGC1a expression levels in lysate of quadriceps proteins (n=4 per genotype). Error bars represent SEM. (B) Western blot analysis of LC3 and p62 expression levels in lysate of TA mitochondrial proteins (n=5 per genotype). Error bars represent SEM. (C) Representative confocal images of myofibres from P100 WT and Drp/MC TA muscles electroporated with pDsRed2-mito and pLC3-YFP plasmids. Scale bar 50um.

**Figure S7.** Polarized mitochondria accumulate close to the fibre nucleus.

Representative confocal image of isolated gastrocnemius myofibres stained with 25nM TMRM for 30 min. Scale bar 20um.

**Figure S8.** mtUPR is not activated in Drp/MC diaphragm and liver.

Western blot analysis of Hsp60 and ClpP expression levels in total lysate of diaphragm and liver of P100 WT and Drp/MC animals (n=6 per genotype). Error bars represent SEM.

**Figure S9.** ER stress response is not detected in adult Drp/MC muscle.

(A) RT-qPCR analysis of Grp78/BiP and Grp94 mRNA levels in quadriceps from P100 WT and Drp/MC mice (n=8 per genotype). (B) RT-qPCR analysis of XBP-1 splicing (n=4 per genotype). Error bars represent SEM.

**Figure S10.** Western blot analysis of representative signalling intermediates of the AKT/mTOR/GSK3 pathway.

Expression levels of Drp1, AKT, P-AKT, S6, P-S6, 4EBP1, P-4EBP1, GSK3β, P-GSK3β in lysates of quadriceps proteins (n=4 per genotype).

**Figure S11.** Reduction of MHC protein synthesis in adult Drp/MC muscle.

Chop (A), MHC (B) and 36B4 (D) mRNA distributions in the fractions collected from the sucrose gradients was determined by RT-qPCR and percentage of transcripts present in polysomal fractions (6-12min) was calculated (n=3 per genotype). (C) Western blot analysis of MHC protein levels (n=6 per genotype). Error bars represent SEM. * P < 0.05, ** P < 0.01 versus wt.

**Figure S12.** GH pathway is downregulated.

RT-qPCR analysis of representative genes of GH pathway in quadriceps from adult WT and Drp/MC mice (n=8 per genotype). Error bars represent SEM. * P < 0.05, ** P < 0.01, *** P < 0.001 versus wt.

**LEGENDS TO SUPPLEMENTARY MOVIES**

**Movie S1**. Three-dimensional reconstruction of mitochondrial network in PhAM TA myofibres from confocal image stacks.

**Movie S2.** Three-dimensional reconstruction of mitochondrial network in Drp/MC/PhAM TA myofibres from confocal image stacks.
