# Supplementary figures and images for: Muscle-specific Drp1 overexpression impairs skeletal muscle growth via translational attenuation
Source: Cell Death Dis. 2015 Feb 26;6(2):e1663–. doi: 10.1038/cddis.2014.595 (PMC4669802; doi:10.1038/cddis.2014.595)

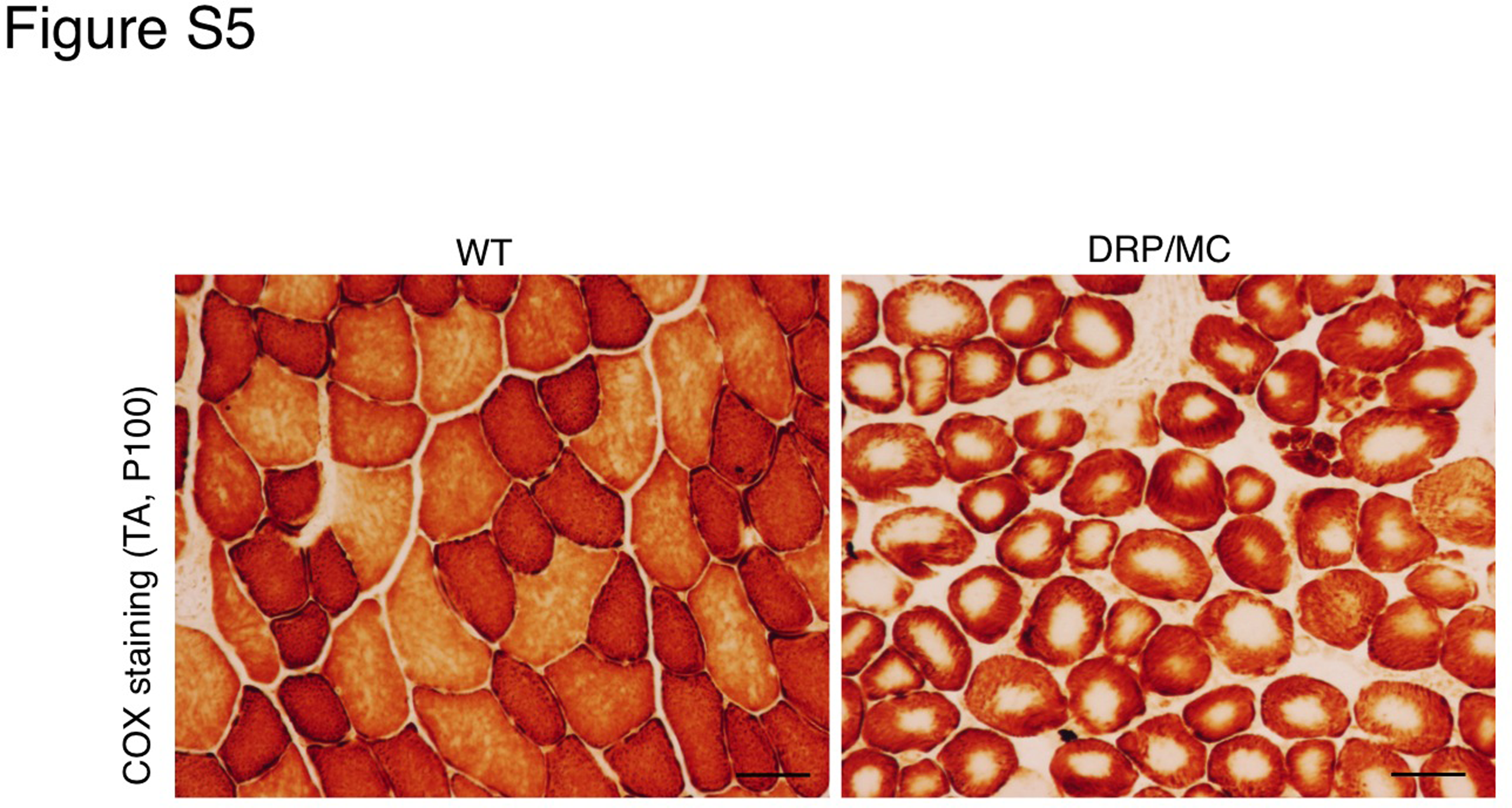

Supplement: Supplementary Figure S5 [file cddis2014595x7.tif]

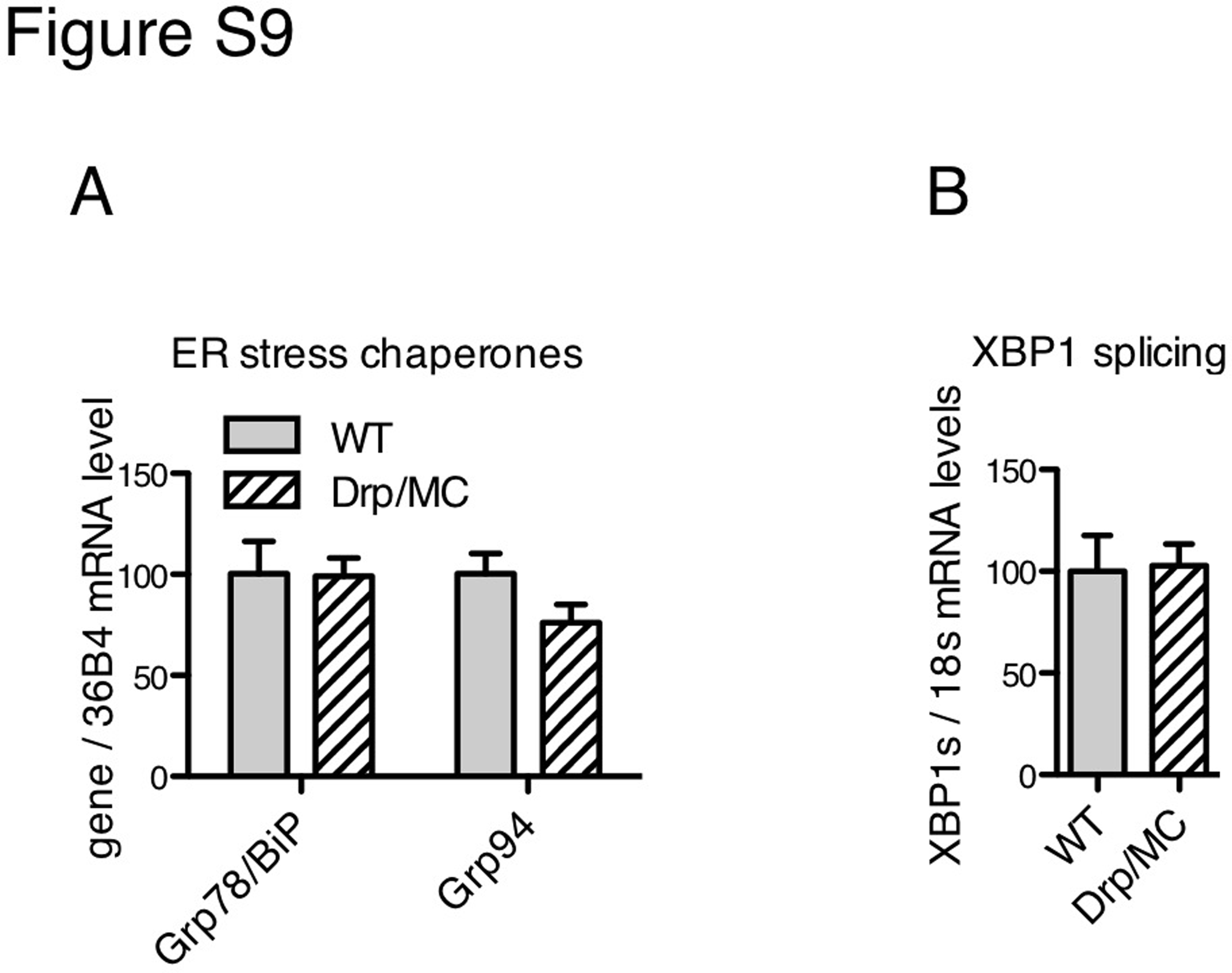

Supplement: Supplementary Figure S9 [file cddis2014595x11.tif]

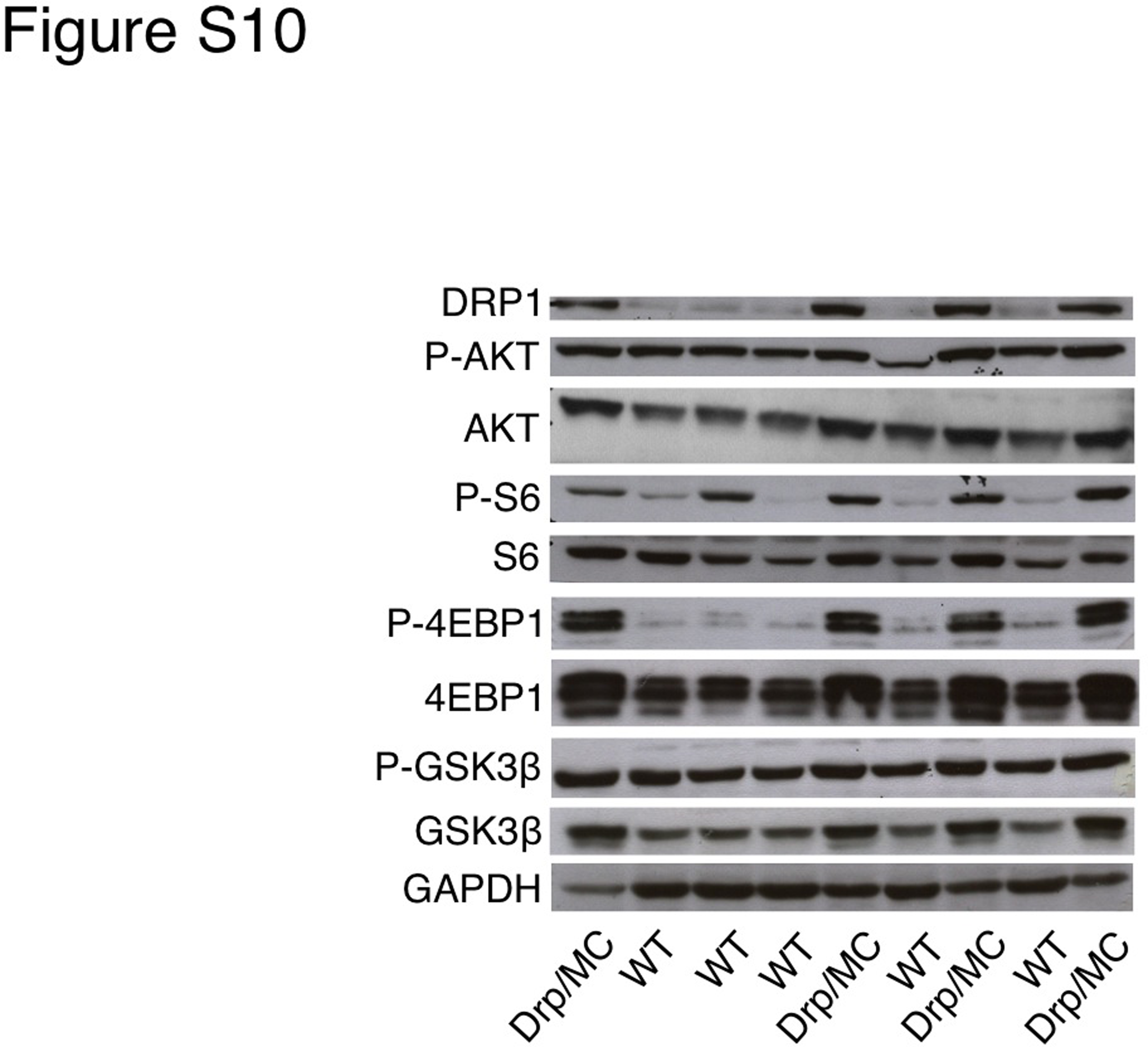

Supplement: Supplementary Figure S10 [file cddis2014595x12.tif]

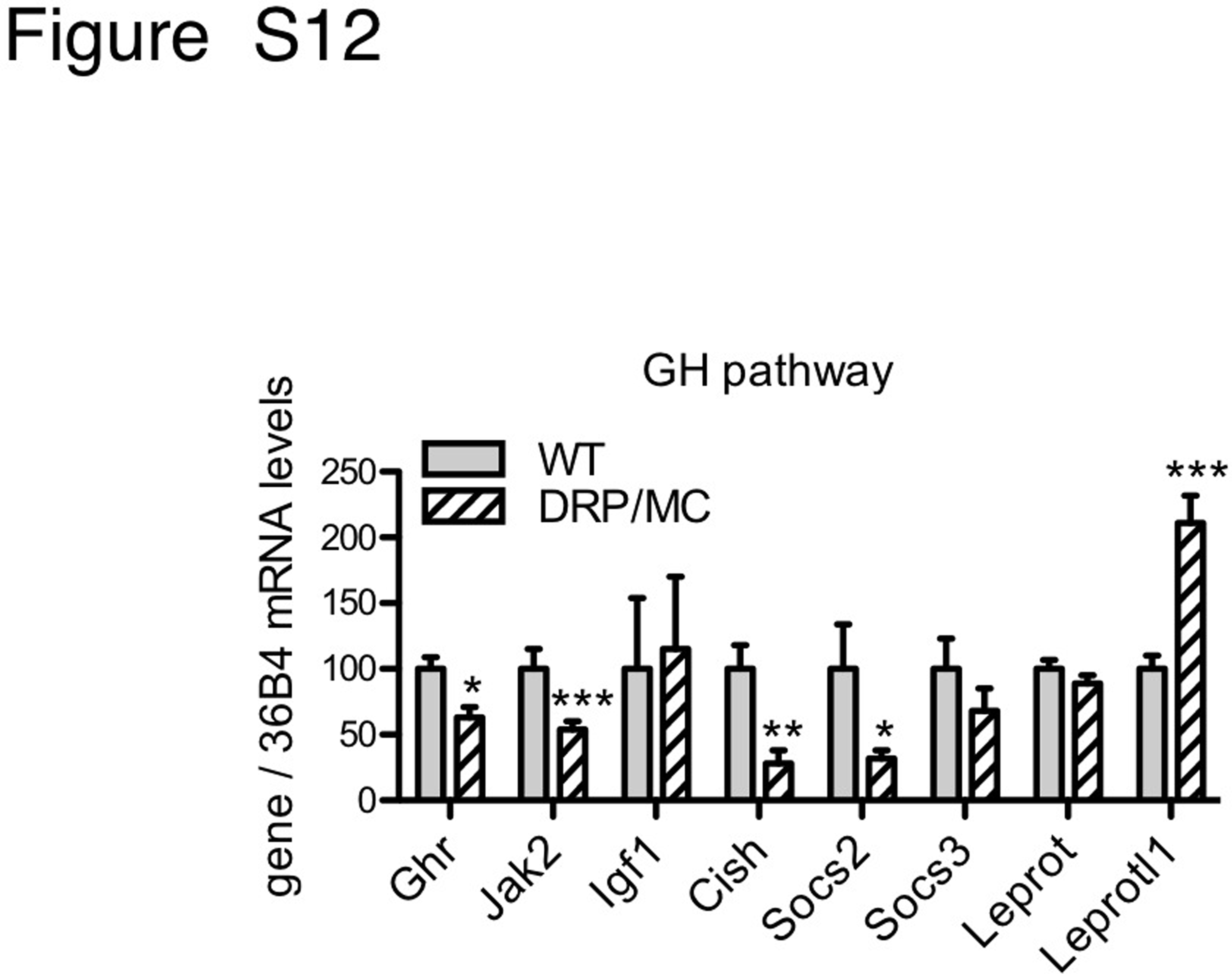

Supplement: Supplementary Figure S12 [file cddis2014595x14.tif]
